# Wireless Soft Scalp Electronics and Virtual Reality System for Motor Imagery‐Based Brain–Machine Interfaces

**DOI:** 10.1002/advs.202101129

**Published:** 2021-07-17

**Authors:** Musa Mahmood, Shinjae Kwon, Hojoong Kim, Yun‐Soung Kim, Panote Siriaraya, Jeongmoon Choi, Boris Otkhmezuri, Kyowon Kang, Ki Jun Yu, Young C. Jang, Chee Siang Ang, Woon‐Hong Yeo

**Affiliations:** ^1^ George W. Woodruff School of Mechanical Engineering College of Engineering Georgia Institute of Technology Atlanta GA 30332 USA; ^2^ Center for Human‐Centric Interfaces and Engineering Institute for Electronics and Nanotechnology Georgia Institute of Technology Atlanta GA 30332 USA; ^3^ School of Computing University of Kent Canterbury Kent CT2 7NT UK; ^4^ School of Biological Sciences, College of Sciences Georgia Institute of Technology Atlanta GA 30332 USA; ^5^ School of Electrical and Electronic Engineering Yonsei University Seoul 03722 Republic of Korea; ^6^ Wallace H. Coulter Department of Biomedical Engineering Parker H. Petit Institute for Bioengineering and Biosciences Institute for Materials Neural Engineering Center Institute for Robotics and Intelligent Machines Georgia Institute of Technology Atlanta GA 30332 USA

**Keywords:** brain–machine interfaces, motor imagery brain signals, virtual reality system, wireless soft scalp electronics

## Abstract

Motor imagery offers an excellent opportunity as a stimulus‐free paradigm for brain–machine interfaces. Conventional electroencephalography (EEG) for motor imagery requires a hair cap with multiple wired electrodes and messy gels, causing motion artifacts. Here, a wireless scalp electronic system with virtual reality for real‐time, continuous classification of motor imagery brain signals is introduced. This low‐profile, portable system integrates imperceptible microneedle electrodes and soft wireless circuits. Virtual reality addresses subject variance in detectable EEG response to motor imagery by providing clear, consistent visuals and instant biofeedback. The wearable soft system offers advantageous contact surface area and reduced electrode impedance density, resulting in significantly enhanced EEG signals and classification accuracy. The combination with convolutional neural network‐machine learning provides a real‐time, continuous motor imagery‐based brain–machine interface. With four human subjects, the scalp electronic system offers a high classification accuracy (93.22 ± 1.33% for four classes), allowing wireless, real‐time control of a virtual reality game.

## Graphical abstract

This image shows a wearable scalp electronic system with virtual reality for motor imagery‐based brain–computer interfaces. The all‐in‐one, portable soft system includes an array of soft microneedles, stretchable interconnectors, and flexible circuits in a wearable platform.

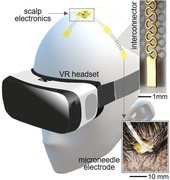

## 1 Introduction

Brain–machine interfaces (BMIs) offer a possible solution for individuals with a physical disability such as paralysis or brain injury resulting in similar motor dysfunction. Among them, a significantly challenging disorder is a locked‐in syndrome, where an individual is conscious but unable to move or communicate.^[^ ^1^ ^]^ Here, BMIs may be able to restore some function to these individuals, providing a greater capability for movement and communication, improving quality of life.^[^ ^1^, ^2^ ^]^ Electroencephalography (EEG) is the premier noninvasive method for acquiring brain electrical activity,^[^ ^3^, ^4^, ^5^ ^]^ where electrodes mounted on the scalp surface record the sum of postsynaptic potentials in the superficial cortex. Conventional research‐ and medical‐grade EEG use a hair cap or a large headgear with multiple rigid electrodes to measure signals at the scalp. These heavy and bulky systems are uncomfortable to wear and often require large, rigid electronics either attached to the system or separated using long lead wires.^[^ ^3^ ^]^ These devices depend heavily on consistent skin–electrode impedances and typically suffer from significant motion artifacts and electromagnetic interferences.^[^ ^3^, ^4^ ^]^ Typically, electrodes are coupled with conductive gels or pastes to improve surface contact and reduce impedance. These interfacial materials are a source of noise due to changes in impedances at these locations due to motion artifacts or material degradation. Overall, conventional systems require extensive setup times and are inconvenient and uncomfortable to use.

Recently, technological advances have allowed for improved signal acquisition through the use of lightweight, flexible electronics and dry electrodes.^[^ ^6^ ^]^ The latest EEG designs display a trend toward wireless, wearable EEG. These are preferable for day‐to‐day mobile EEG monitoring, with compact, battery‐powered designs over conventional amplifiers and hair‐cap EEG. For mobile systems, dry electrodes are preferred due to short setup times, no skin irritation, and excellent long‐term performance.^[^ ^7^, ^8^ ^]^ In addition, they often perform better than gel‐based EEG sensors while providing long‐term wearability without reduced signal quality.^[^ ^4^, ^7^, ^9^ ^]^ Recent developments in skin‐interfaced electrodes for biopotential acquisition demonstrate new strategies and solutions for on‐skin bioelectronics.^[^ ^10^ ^]^ Examples include the use of screen‐printed highly conductive composites^[^ ^11^ ^]^ and nanowire‐based networks fabricated via interfacial hydrogen bonding in solution ^[^ ^12^ ^]^ with excellent stretchability and interfacial conductive properties. There is a multitude of strategies for noninvasive EEG BMI paradigms.^[^ ^13^ ^]^ In our prior work,^[^ ^3^ ^]^ steady‐state visually evoked potentials (SSVEPs) were studied, where subjects can operate a machine interface by shifting their gaze between flickering stimuli of differing frequencies. However, with the recording of SSVEPs, practical applications are limited due to the requirement of an array of visual stimuli impeding the operator's view. Also, we found out that the bright, flickering stimuli caused eye strain and fatigue when used for extended periods. Alternatively, motor imagery (MI) is a greatly advantageous paradigm for persistent BMI as it does not require the use of external stimuli; its classes are based on imagined motor activities such as opening and closing a hand or moving feet.^[^ ^14^, ^15^ ^]^ With MI, specified motor imagery tasks results in sensorimotor rhythm fluctuations in the corresponding motor cortex region, which are measurable with EEG.

Here, we introduce fully portable, wireless, soft scalp electronics (referred to as “SSE”), composed of three major components: 1) multiple flexible microneedle electrodes for mounting on the hairy scalp, 2) laser‐machined stretchable and flexible interconnects, and 3) a low‐profile, flexible circuit. Additionally, the inclusion of a virtual reality (VR) component allows for a convenient and immersive training environment to assist with motor visualization. These components form a monolithic EEG system optimized toward minimizing artifacts and maximizing signal quality. Epidermis‐penetrating electrodes offer optimal impedance density on the scalp, improving signal‐to‐noise ratio and spatial resolution for MI recording. Overall, this study demonstrates a feasible approach to a high‐performance BMI system integrating a powerful machine‐learning algorithm and virtual reality. The imperceptible, hair‐wearable system with only 6 EEG channels results in high accuracy of 93.22 ± 1.33% for four classes with a peak information transfer rate of 23.02 bits/min with four human subjects. The SSE demonstrates an accurate control of a virtual reality game using a MI paradigm.

## 2 Results and Discussion

### 2.1 Overview of a Wireless Soft Scalp Electronic System for MI‐Based BMIs

**Figure 1:**
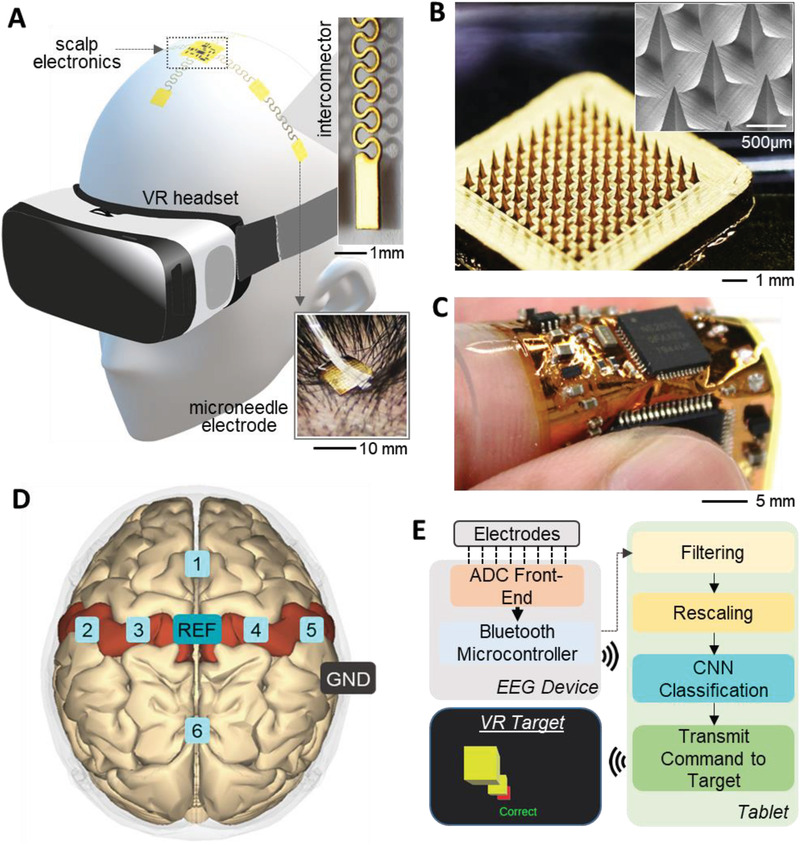
Overview of the wireless scalp system for motor imagery brain signal detection, featuring fully portable electronics, stretchable interconnectors, and flexible microneedle arrays. A) An illustration of a subject wearing a VR headset and scalp electronics with a close‐up of stretchable interconnectors (top‐right photo) and a flexible microneedle electrode (bottom‐right photo). B) A zoomed‐in photo of an array of microneedles along with a magnified SEM image of the needles (inset). C) A picture of a flexible wireless circuit with integrated chips, showing mechanical compliance of the membrane circuit. D) Locations (top view) of the microneedle electrodes for classification of motor imagery brain signals, including six recording channels, one reference (REF) and one ground (GND). E) A flow chart describing the entire data processing sequence from EEG recording to the control of targets in a virtual reality system via a machine learning classification algorithm (convolutional neural network, CNN).

**Figure** **1** summarizes an overview of an SSE for an MI brain signal detection and a persistent BMI. The fully integrated, portable, wireless wearable system enables long‐range, real‐time, and continuous recording of brain signals to accurately classify MI with a virtual reality (VR) headset for immersive visualization training. An illustration in Figure 1A captures the entire system with a subject wearing a VR headset, and an SSE with an array of integrated stretchable interconnectors bonded between flexible microneedle electrodes (FMNEs) and the central circuitry. The wearable soft headset is composed of low‐modulus elastomeric bands, molded together for securing multiple FMNEs in place (Figure S1, Supporting Information). The primary band wraps around the head about the axial plane with an attached membrane crossing from the forehead to the inion to secure the electrodes along that axis, while another membrane from ear to ear secures the electrodes on the temporal lobes.

An optical photo in Figure 1B shows an array of each FMNE, while the inset shows a scanning electron microscope (SEM) image of the needles' geometry. Each high‐aspect‐ratio needle has 800 µm in height with a base width of 350 µm, which allows a successful penetration of the stratum corneum to offer a significantly decreased contact impedance. An array of FMNEs is constructed via a microreplica‐molding process on polyimide, followed by a coating of thin‐film conductive metals (Cr and Au). Details of the fabrication process appear in Section S1 and Figure S2 in the Supporting Information. An additional set of photos in Figure S3 in the Supporting Information shows a positive epoxy mold, negative silicone mold, and the outcome of FMNEs coated with metals. The polyimide substrate is thin enough to allow electrode flexion to better conform to the scalp surface. The area of each electrode set is about 36 mm^2^, which could improve the EEG spatial resolution over the conventional, large cup electrodes (100 mm^2^) that also require conductive gels. The SSE in Figure 1A includes a flexible wireless circuit, placed on top of the elastomeric band on the crown of the head for maximized isolation from the body. A fabricated soft circuit in Figure 1C demonstrates high mechanical flexibility when mounted on an index finger. The device fabrication process is detailed in Figure S4 (Supporting Information). The flexible wireless circuit maintains functionality even with an excessive bending up to 180 degrees with a radius of curvature of 1.5 mm, which is supported by both computational and experimental studies (Figure S5A–C, Supporting Information). More importantly, this device shows robust mechanical reliability from an experimental study with 100 bending cycles, quantitatively measured by the change of electrical resistance (Figure S5D,E, Supporting Information). A robust connection between the circuit and multiple FMNEs is enabled by stretchable interconnectors (details of the fabrication process in Section S2 and Figure S6 in the Supporting Information). A combination of metallization, laser‐cutting process, and transfer‐printing step allows fabricating reliable membrane interconnectors. A schematic illustration in Figure 1D shows the optimized locations of FMNEs based on the analysis of publicly available MI data and experimental results. Details of the optimized selection of six channels appear in Section S3 and illustrated in Figure S7 in the Supporting Information. We speculated that a smaller number of channels than the conventional EEG setup is possible due to the improved skin–electrode contact from the FMNEs. The approximate positions of the electrodes corresponding with the standard 10–10 electrode placement system ^[^ ^16^ ^]^ are Fz, C5, C3, C4, C6, and POz, with the reference electrode at Cz, and the ground electrode placed at the mastoid (Figure S8, Supporting Information). This setup is optimized for capturing event‐related synchronization and desynchronization relating to separate hands and both feet, as well as capturing overall alpha rhythm activity. The system setup was determined with the aim of reducing the number of electrodes and setup complexity without significant sacrifices in classification performance. A flowchart of the overall setup and data flow are shown in Figure 1E, toward control of a VR game via real‐time MI data.

### 2.2 Mechanics and Characterization of Stretchable Interconnectors and Microneedle Electrodes

**Figure 2:**
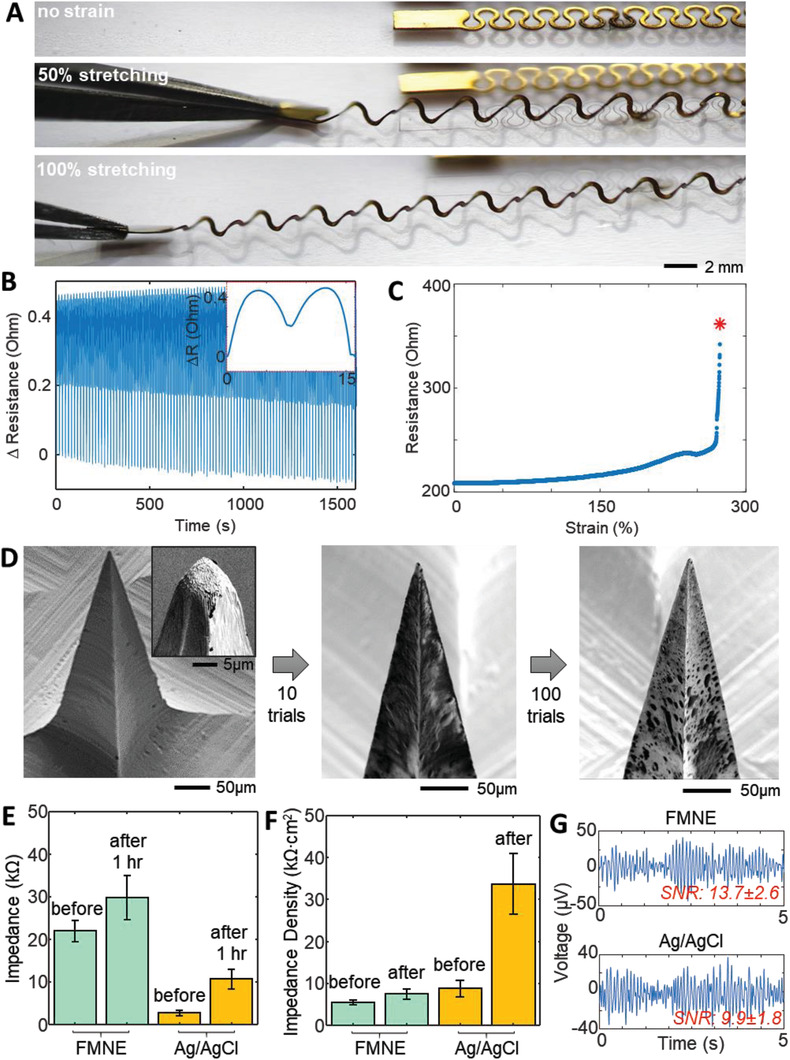
Characterization of stretchable EEG interconnectors and flexible microneedle electrodes (FMNE). A) Photos of stretching of a stretchable EEG interconnector up to 100%. B) Electrical measurement of resistance change of the interconnector with 60% strain for 100 cycles, showing negligible changes in resistance. The inset shows the resistance change over a single stretch cycle. C) Measurement of electrical resistance change of the interconnector, showing mechanical fracture after 250% of tensile stretching. D) A series of SEM close‐up images microneedle electrode tip: unused (left, inset: zoom‐in view of the tip), after ten insertions into porcine skin (middle), and after 100 insertions into the tissue (right). E) Skin–electrode contact impedance that compares the performance between FMNE and conventional Ag/AgCl cup electrodes (error: standard deviation with *n* = 4 subjects). F) Calculated impedance density from data (E), showing a dramatic increase of the impedance change from the conventional electrode. G) SNR comparison of EEG alpha rhythms measured by FMNE (top) and conventional Ag/AgCl electrodes (bottom).

**Figure** **2** summarizes the results of fabricated stretchable interconnectors, mechanical characterization, FMNE's structural robustness, and a comparison of skin–electrode contact impedance between FMNEs and conventional metal/gel electrodes. A set of photos in Figure 2A captures a highly stretchable property of a fabricated interconnector embedded in the SSE. With the help of an out‐of‐plane buckling, the thin‐film connector, enclosed by a low‐modulus elastomer, shows mechanical reliability upon cyclic stretching when measured by a digital force gauge (EMS303, Mark‐10) and a multimeter (DMM7510, Tektronix). Details of the mechanical testing method appear in Materials and Methods. The result in Figure 2B shows the change of electrical resistance of the interconnector in Figure 1A with an applied tensile strain of 60% and 100 cyclic loadings; each full cycle takes  ≈15 s (Figure S9, Supporting Information). The level of resistance fluctuation during cycles was very consistent with less than 60 mΩ change, and the total resistance change after 100 cycles is about 70 mΩ. In addition, the interconnector's end‐to‐end resistance was monitored during a maximum strain test. As shown in Figure 2C, the stretchable connector shows a yielding after 200% strain, which is eventually fractured at ≈275% strain. Close‐ups of the interconnect wire at the maximum strain in Figure S10 in the Supporting Information captures the failure point close to the contact pads. Overall, the presented data set demonstrates a reliable mechanical performance of the stretchable interconnector that integrates multiple sensors with a wireless circuit in the SSE. The maximum stretchability is well beyond what would be realistically expected from the scalp‐wearable application.^[^ ^3^, ^4^ ^]^

Additional sets of experimental studies in Figure 2D–G and Figures S11 and 12 in the Supporting Information summarize the mechanical and electrical characteristics of a fabricated FMNE. High‐resolution, close‐up SEM photos (Figure 2D) capture the terminal end of a fabricated FMNE before and after tissue insertion. In this study, a porcine skin was used to test the mechanical robustness with 100 insertions. Aside from cosmetic blemishes due to porcine tissue fluids, the FMNE tips “shape and electrical coating remained intact. A quantitative mechanical test in Figure S11 in the Supporting Information validates the SEM observation by measuring the required fracture force in buckling. A total of five fabricated FMNEs could withstand an averaged applied force up to 626 mN, which is well above the skin insertion force (20–167 mN) of a single microneedle.^[^ ^17^ ^]^ Another mechanical test of cyclic bending of FMNEs (Figure S12, Supporting Information) supports the mechanical robustness in tissue insertion. The needle electrode was continuously bent up to 100 times with a radius of curvature of 5 mm while measuring the change of electrical resistance. The result shows a negligible resistance shift of less than 0.6 Ω. The prior works ^[^ ^18^ ^]^ proved that gold‐based electrodes, mounted on the skin surface, are safe to use due to their excellent biocompatibility. In this work, we conducted an additional experimental study to validate the safety of skin‐penetrating electrodes, FMNE, via a cytotoxicity test (details in Figure S13 in the Supporting Information). A 48 h test on human skin demonstrated minimal skin immune reaction, showing skin redness fading after 1 h (details in Figure S14 in the Supporting Information). Human keratinocytes were seeded on the electrodes and cultured for over 5 days. For the immunofluorescent labeling, cells were fixed and incubated with Alexa Fluor 555 Phalloidin and Hoechst for 2 h, and the cytotoxicity was quantified with a PrestoBlue Cell Viability reagent. The summarized results show that the difference in cell densities and morphologies between the control and a gold‐coated FMNE is negligible. Also, the measured cytotoxicity between the two samples shows no significant difference, capturing the electrodes” safety for human study.

For a high‐quality recording of brain signals, the impedance between the electrode and skin should be low, demonstrating an intimate contact. Two types of electrodes (FMNE and conventional Ag/AgCl) were utilized in this study by mounting both on human subjects' scalps. As summarized in Figure 2E,F, the FMNE could maintain the skin–electrode contact impedance levels even after 1 h of continuous use in EEG detection. The impedance density (ID, kΩ cm^2^) was calculated by using the measured impedance (*Z*, kΩ) and contact area (*A*, cm^2^): ID  =  *Z x* *A*. To prove the enhanced performance of the FMNE in EEG recording, two types of electrodes were used to measure signals on POz and Cz for 2 min simultaneously. The recorded alpha rhythms (Figure 2G) show an average signal‐to‐noise ratio (SNR) of 13.7 ± 2.6 dB (top graph) and 9.9 ± 1.8 dB (bottom graph) for a dry FMNE case and a wet Ag/AgCl cup electrode with conductive paste, respectively. Overall, the skin‐penetrating FMNE shows more reliable, higher performance signal recording than the conventional, gel‐based metal electrode.

### 2.3 Preprocessing and Classification of MI Brain Signals with Convolutional Neural Networks (CNNs)

Recent studies in multichannel MI EEG classification suggest that using CNNs on preprocessed time‐domain data provides the most accurate results.^[^ ^19^, ^20^, ^21^ ^]^ For validation of the machine‐learning performance, we compared CNNs with conventional feature‐extraction methods via power spectral density analysis (PSDA) and support vector machines (SVM). Two types of spatial CNN (Figure S15A, Supporting Information) and traditional CNN (Figure S15B, Supporting Information) methods were also compared since spatial convolutions to classify multiclass MI data were introduced by a recent study (17). We used data recorded from four subjects using FMNEs, preprocessed via a bandpass filter (third‐order Butterworth, 4–33 Hz). The spatial CNN method achieved very high accuracy (93.22 ± 1.33%), whereas the standard CNN performed poorly in direct comparison (68.51 ± 3.89%), as shown in Figure S15C,D (Supporting Information). This result suggests that the spatial‐CNN method's modified architecture is better at extracting spatial information features from the multichannel MI data. Regarding feature extraction, PSDA demonstrates event‐related desynchronization (ERD) and event‐related synchronization (ERS) across MI classes when assessing six‐channel data (Figure S16, Supporting Information). However, not all ERS/ERD events are apparent in this data, suggesting more subtle features obscured within the time‐domain data. Additional results in Table S1 in the Supporting Information show that PSDA performs very poorly when compared with filtered time‐domain data.

**Figure 3:**
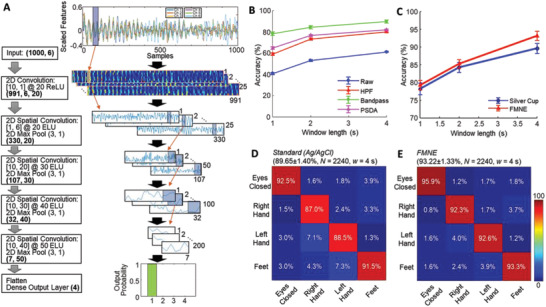
Preprocessing and classification of motor imagery brain signals with CNNs. A) Detailed illustration of a spatial CNN model with hidden layers of brain signals acquired from six EEG channels. This model demonstrates the capability of decomposing spatial features from multiple dipolar sources of the motor cortex. B) Comparison of spatial‐CNN classification accuracy of four cases, including raw data, high‐pass filtered data (HPF), bandpass‐filtered data (Bandpass), and power spectral density analysis (PSDA) across multiple window lengths (1, 2, and 4 s). Error bars show a standard error from four subjects. C) Comparison of spatial‐CNN classification accuracy between the conventional Ag/AgCl gel electrodes and the newly developed FMNE across multiple window lengths (1, 2, and 4 s). Error bars show a standard error from four subjects. D) A confusion matrix representing results from the real‐time accuracy test of motor image brain data, acquired by conventional Ag/AgCl electrodes, with an overall accuracy of 89.65% (*N* = 2240 samples, window length = 4 s, and four human subjects). E) A confusion matrix representing results from the real‐time accuracy test of motor image brain data, acquired by FMNE, with an overall accuracy of 93.22% (*N* = 2240 samples, window length = 4 s, and four human subjects).

**Figure** **3** shows a summarized set of preprocessing and classification of MI data with CNNs. An illustration in Figure 3A shows a spatial CNN model's details with hidden layers of brain signals acquired from six EEG channels. This model demonstrates the capability of decomposing spatial features from multiple dipolar sources of the motor cortex. The comparison data in Figure 3B shows the different accuracies between three basic preprocessing methods and one with PSDA. Details on the PSDA preprocessing are provided in the Experimental Section. In this comparison, the spatial‐CNN with filtering shows better accuracy than PSDA. The results in Figure S17 in the Supporting Information compare the classification results with PSDA and SVM with a cubic kernel function on conventional Ag/AgCl electrodes and FMNEs, showing a better accuracy from the FMNE dataset (4 s windows). The graph in Figure 3C compares the spatial‐CNN method's classification accuracy between the conventional Ag/AgCl electrodes and the newly developed FMNEs across multiple window lengths (1, 2, and 4 s). Error bars show a standard error from four subjects. All data sets that compare the performance between Ag/AgCl electrodes and FMNEs appear in Figure S18 (Supporting Information). Two confusion matrices in Figure 3D–E compare the performance of Ag/AgCl electrodes and FMNEs in classification accuracies with four classes. The real‐time accuracy test of motor image brain data shows an overall accuracy of 89.65% and 93.22% for Ag/AgCl and FMNEs, respectively (2240 samples, window length = 4 s, and four human subjects). Overall, the outstanding performance of EEG‐based MI classification with the SSE exceeds the results from prior works (**Table** **1**).

**Table 1:** Performance comparison between the SSE and reported values for EEG‐based MI classification

Reference	Year	Accuracy [%]	No. of electrodes	No. of classes	Length (s)	No. of subjects	ITR [bits min^−1^]
This work (SSE)	2020	93.22 ± 1.33	6	4	4	4	23.02 ± 1.11
[15]	2019	83.0	22	4	4	9	16.09^a)^
[20]	2017	86.41 ± 0.77	28	2	3	2	8.53 ± 0.42^a)^
[25]	2016	77.6 ± 2.1	3	2	2	9	6.98 ± 1.18^a)^
[26]	2016	84.0	3	2	2	9	10.97^a)^
[27]	2019	95.4	128	4	2	9	49.74^a)^
[21]	2017	84.0	44	4	4	9	16.68^a)^

^a)^ Values calculated from the reported data; they do not consider potential interface latency.

### 2.4 VR Implementation for MI Training and Video Game Demonstration

**Figure 4:**
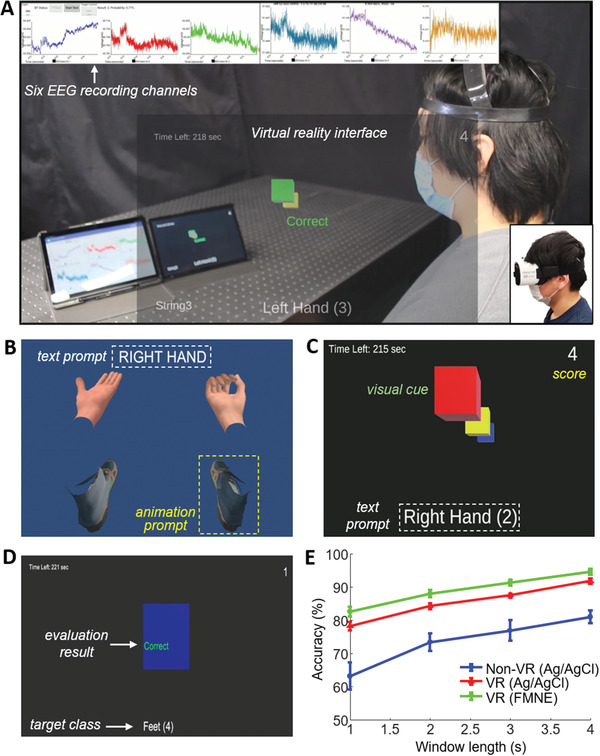
Virtual reality (VR) implementation for motor imagery training and real‐time control of a video game demonstration. A) An overview of a study setup, including a subject wearing the SSE, real‐time EEG data measured from six electrodes (top inset), an example of a VR interface (middle inset), and a photo of a subject wearing a VR headset (bottom‐right inset). Examples of the training and testing processes of a VR game. Details appear in Video S1 and S2 (Supporting Information). A video clip of a subject demonstrating highly accurate video game performance is shown in Video S3 (Supporting Information). B) A modified view of VR visuals provided to test a subject with text and animation prompts. C) A video game interface designed for MI response testing with clear color‐coded visual cues as well as a text prompt. D) An example of evaluation output according to the target class. E) An accuracy comparison between non‐VR setup and VR setup (two types of electrodes) classified with spatial‐CNN model, demonstrating the superior performance of VR as a training implement (*n* = 2240 samples from four subjects, 560 samples per subject, window length *w* = 4 s).

The combination of the high‐performance SSE and the CNN‐based machine learning allows for seamless integration of the system with a VR interface. The experimental study in **Figure** **4** shows the result of a VR implementation for MI training and real‐time control of a video game. An overview of a study setup in Figure 4A captures a subject wearing the SSE, real‐time EEG data measured from six electrodes (top inset), and an example of a VR interface (middle inset). A customized Android application provides real‐time, continuous motoring of 6‐channel MI signals (details of the interface in Figure S19 in the Supporting Information). Examples of the training and testing processes of a VR game are shown in Figure 4B–D; a modified view of VR visuals is given to a subject with text and animation prompts. A video game interface, designed for MI response testing, shows clear color‐coded visual cues as well as a text prompt. Additional details of the training and testing procedures appear in Videos S1 and S2 (Supporting Information). In addition, a video clip of a subject demonstrating highly accurate video game performance (accuracy: ≈95%) is shown in Video S3 (Supporting Information). The summarized set of graphs in Figure 4E shows an accuracy comparison between non‐VR setup and VR setup (two types of electrodes) classified with the spatial‐CNN model. The result demonstrates superior performance of VR as a training implement (2240 samples from four subjects, 560 samples per subject, window length *w* = 4 s). At the same time, additional accuracy improvement is observed with the FMNE and VR setup. The enhanced accuracy would come from an immersive VR program with disembodied hands and feet shown within the subject's field of view in approximately the same position as their existing limbs. During the study, a subject could gently rotate or tilt their head to glance at the hands or feet as their focus requires.

## 3 Conclusion

The presented research demonstrates a highly accurate classification of a four‐class, MI‐based BMI with as few as six channels. The SSE system provides a competitive accuracy and information transfer rate (ITR) with low response times (93.22 ± 1.33% for 4 s of data; ITR = 23.02 ± 1.11 bit min^−1^). This study also shows a subject playing a rhythm‐type video game where they must perform MI tasks in a specific prompted order within specific timing intervals to score points. Using 4 s windows, subjects could achieve high scores, only missing a few points per 5 min game session. Subjects indicated that controlling the interface required a minimal effort on MI visualization.

The excellent signal reproduction with minimal artifacts may be attributed to the monolithic and compliant nature of the SSE. In conventional systems with rigid electronics and inflexible wiring, motion causes stress concentration at the skin–electrode interface. These stresses, when combined with conventional gel‐based electrodes, cause significant skin–electrode impedance variations, resulting in motion artifacts. Where dangling wires are involved, the influence of gravity compounds these issues. The newly developed FMNEs in the SSE provide an improved SNR by penetrating through the most superficial skin layers composed of dry and dead skin cells. By penetrating these layers and placing the conductive portion of the electrodes well within the dermis, we could significantly reduce the impedance density while allowing for smaller electrodes than the conventional setting and improving spatial resolution for MI detection. When compared head‐to‐head against the gold‐standard Ag/AgCl gel electrodes, the FMNE achieved superior SNR. At the start of data acquisition, Ag/AgCl electrodes with abrasive gel to remove the superficial dead skin layer were favored. However, with the gel degrading over time, and as the impedance density and classification results show, the FMNE outperformed the conventional electrodes in every respect.

Additionally, an optimized training configuration in the form of an immersive VR environment resulted in a vast improvement in the system performance. The VR environment appears to have allowed subjects to visualize MI tasks better and easily focus on the training process. As a result, VR data is of higher quality, achieving superior classification performances (Table 1). Without the visual cues from the VR headset, typically, MI signals could only be sustained for a few seconds at a time purely from imagination. With the VR headset, it allows the experimenters to record continuous, high‐quality MI activity from one class for a minute at a time and could continue for an indefinite amount of time. Overall, the VR‐based data yielded much higher quality data, resulting in optimal classification outcomes. Even after removing the headset, subjects could maintain excellent accuracy in test demonstrations, as shown in the video game demonstration.

As presented in this work, MI detection is advantageous in BMI paradigms since MI signals can be spontaneously produced by individuals, unlike with evoked potentials. Therefore, MI offers more significant potential as a general‐purpose interface. Currently, the main limitation is reconstructing poor‐quality signals from the scalp to interpret motor cortex activity. Future work would focus on further optimizing electrode placement toward maximizing the number of functional MI classes while still maintaining high accuracy. Additionally, the integration of additional paradigms will be considered to add more classes and functions for persistent BMIs.

## 4 Experimental Section

### Mechanical Characterization of Stretchable Interconnectors

To study the interconnector's stretchability, 2 in. long test specimens were fabricated with the same materials and fabrication processes as the final versions used in the headset. The test specimen was fixed to a motorized test stand (ESM303, Mark‐10) for its mechanical stretching. The change in resistance of the interconnector during the elongation was observed with a multimeter (DMM7510, Tektronix). The first test specimen was stretched up to its mechanical failure to follow its maximal stretchability. The second specimen was used for the cyclic stretching test with 100 cycles with 60% stretch to verify its reliability under repeated use within the range of actual stretchability needed.

### Experimental MI Classes

Three MI tasks were included in the experimental classes as well as alpha rhythms (the subject has eyes closed) for a total of four classes toward controlling the interface. Alpha rhythms were used as a null class in tasks where the subject wanted to pause control over some target. For the non‐VR examination, subjects were asked to imagine the actions of opening and closing their hands, as well as depressing a pedal with both feet in the first person for the tasks. With the VR examination, the subjects were provided with clear visual guidance on what they should be imagining, using animated disembodied limbs within the normal field of view that a subject could view their own limbs.

### Cytotoxicity Study

To observe cell morphologies on the sensors, the electrodes were attached at the bottom of the 8‐well slide (Nunc Lab‐Tek II Chamber Slide System). Human keratinocytes (ATCC, PCS‐200‐011) were seeded onto the electrodes and cultured with a keratinocyte growth kit (ATCC, PCS‐200‐040) for over 5 days. For the immunocytochemistry, cells were fixed and blocked with blocking buffer (2% BSA, 0.5% Goat serum, and 0.5% Triton X‐100 in PBS). The cells were then incubated with Alexa Fluor 555 Phalloidin and Hoechst for 2 h, followed by 3 times of PBS wash. Slide wells were removed, and the cells were covered with cover glass to image under a fluorescent microscope. The cytotoxicity test was performed using the PrestoBlue Cell Viability reagent following the protocols provided by the manufacturer. The keratinocytes cultured for 5 days on the sensors were treated with PrestoBlue reagent, diluted tenfolds with DMEM, for 10 min in a 37 °C CO_2_ incubator. The cytotoxicity of the cells was detected using fluorescence measurement (excitation 569 nm and emission 586 nm).

### Recording of MI Data with Human Subjects

When recording with conventional Ag/AgCl electrodes, each subjects' skin was cleaned by gently rubbing with an alcohol wipe, and dead skin cells were removed using an abrasive gel in order to maintain a contact impedance below 10 kΩ on all electrodes. The abrasive gel was removed using an alcohol wipe and the surface dried using a clean paper towel. On the other hand, for the FMNEs, the only skin preparation conducted was a gentle rub of the electrode location with an alcohol wipe. The EEG data were recorded using a custom application running on an Android Tablet (Samsung Galaxy Tab S4), using Bluetooth Low Energy wireless communication. This study was conducted by following the approved IRB protocol (#H19553) at the Georgia Institute of Technology.

### Preparation of Training and Testing Data

For all experiments, data were sampled at 500 Hz, and down sampled to 250 Hz for analysis and classification. Training data involve continuous recording of a single task for 1 min. The subject begins with their eyes closed to target alpha rhythms. After 1 min, an auditory cue lets the subject know to open their eyes; then, they may watch for visual cues to follow the MI instructions. Two seconds of training data were discarded between each class to allow the subject time to react to the new cues. For all four classes, this process takes 4 min per trial, and the process is the same with and without the use of a VR headset. To train the SVM and CNN models, training data were subdivided into window sizes of 1, 2, and 4 s to gauge accuracy over multiple window sizes, with 50% overlap between consecutive windows. There are approximately 112 samples (4 s) per trial for the training set for a total of 560 samples per subject. Therefore, there are 2240 samples for all four subjects.

### Feature Extraction and Classification

Data analysis involved power spectral density estimation using the Welch method,^[^ ^22^ ^]^ as described in prior work.^[^ ^3^ ^]^ Here, the data is down sampled to 250 Hz, then preprocessed using a third‐order Butterworth bandpass filter from 4 to 33 Hz and segmented into 4 s windows with a 50% overlap between consecutive windows. Power spectral density estimation is then performed using 512‐point overlapping Hann windows. This PSDA data is then trained using an SVM with a Cubic polynomial kernel function. For all CNN‐based classifiers, the data is simply preprocessed using a third‐order Butterworth bandpass filter from 4 to 33 Hz and segmented into 4 s windows with a 50% overlap between consecutive windows.

### Cross‐Validation of Classification Algorithms

A fivefold cross‐validation scheme was used in all classification models, with four recordings being used for training and the fifth used for validation, across all five recordings. For the CNN, the batch size used was 64, and the training was run for 200 epochs or aborted early if the classification of the validation data did not improve for 20 epochs.

### BMI Control Scheme

The previously mentioned custom Android application was designed to classify 4 s windows of EEG data using the selected subject's optimal classifier. The classifications occurred every 0.16 s, and two consecutive classifications which agreed were considered a confirmed instruction and transmitted to the target. The Android application transmits commands to the VR video game interface via Bluetooth Classic and can connect to multiple other targets, including vehicles and communication interfaces.

### Calculation of ITR

BMI performance is assessed using an ITR metric, measured in bits per minute. ITR is calculated based on the number of classes, the time required for each command, and the accuracy of the system and is calculated as follows

(1) $$\mathrm{ITR}\;=\left(\log_{2}N+A\log_{2}A+\left(1-A\right)\log_{2}\left(\frac{1-A}{N-1}\right)\right)\;\times \left(\frac{60}{w}\right)\frac{\mathrm{bits}}{\min}$$

where *N* is the number of targets, *A* is the accuracy, and *w* is the combined length of time required to execute a command, including data acquisition time plus processing, classification, and execution latencies.

### Calculation of SNR

To compare SNR between FMNEs and conventional Ag/AgCl cup electrodes, two healthy subjects were selected, and scalp locations POz and Cz were given a light rub with alcohol cleaning wipes. Sterile FMNEs were placed at each location, with Ag/AgCl electrodes applied with conductive paste as close as possible without touching. The paste contact area was maintained at ≈100 mm^2^, as to not affect impedance dramatically between tests. Signals were monitored using the SSE EEG, configured for two bipolar channels at a sampling rate of 500 Hz. The subject was asked to close their eyes, and alpha rhythms were recorded for 2 min. The data were filtered using a third‐order Butterworth high‐pass filter, with a cutoff frequency of 4 Hz, then were segmented into 5 s windows (2500 points), and analyzed using power spectral density estimation via the Welch method,^[^ ^22^ ^]^ using 4096 Hann window for bias reduction. The amplitude of the alpha rhythm signal was extracted using MATLAB's built‐in *findpeaks* method, and the noise was assumed to be the peak amplitude outside the main peak, and the SNR was calculated as follows^[^ ^23^ ^]^

(2) $$\mathrm{SN}R_{\mathrm{dB}}=\;10\log_{10}\left[{\left(\frac{A_{\mathrm{signal}}}{A_{\mathrm{noise}}}\right)}^{2}\right]$$

### VR Application

The Unity (version 2018.4) software was used to create the VR application for use with the Samsung Gear VR (connected to a Samsung S7). The 3D models of the hands and feet were created and animated through the use of Maya version 2018. The VR application utilizes Bluetooth technology to exchange data with the controller application on the tablet pc. When the controller application detects an action such as “right‐hand movement” or “eyes closed” from the user EEG signal, the application will send a byte array corresponding to that action through the connected Bluetooth channel to the VR application to inform them of the player action. In the VR game, cubes with four different colors (Blue, Green, Red, Yellow) will be generated, each corresponding to an action (feet movement, left‐hand movement, right‐hand movement, eyes closed) that is required from the users. The game lasts a total of 240 s. After the initial 1 s, the game will spawn a new cube every 4 s. The users need to provide the correct action through the EEG sensors in response to the color of the cube. For example, for a green cube, the user needs to move their left hand. When they perform a correct action, a green “correct” text will be displayed, and the cube will disappear, and the user will receive 1 point. For a wrong action, a red‐colored “Wrong” text will be shown as feedback.

### Statistical Analysis

Digitized EEG input data is segmented into windows of appropriate length (1–4 s), and bandpass filtered (third‐order Butterworth, 4–33 Hz) before linear upscaling, so that the min and max value of the recording is [−1, 1]. Result data is presented as mean ± SEM, where SEM is the standard error of the mean. The sample size for each subject was *N*_subject_ = 560, with a total number of samples of *N* = 2240. Data were processed in MATLAB (Mathworks), and machine learning training and evaluation were completed using an open source machine learning software library (Tensorflow v2.4.0, Google, Inc.).^[^ ^24^ ^]^

## Conflict of Interest

Georgia Tech has a pending patent application related to the work described here.

## Supporting information

Supporting Information

Supplemental Video 1

Supplemental Video 2

Supplemental Video 3

## Data Availability

Data can be shared upon request.
