# Supplementary material for: Wireless Soft Scalp Electronics and Virtual Reality System for Motor Imagery‐Based Brain–Machine Interfaces
Source: Adv Sci (Weinh). 2021 Jul 17;8(19):2101129. doi: 10.1002/advs.202101129 (PMC8498913; doi:10.1002/advs.202101129)
Supplement: Supplementary file 1 — Supporting Information [file ADVS-8-2101129-s003.pdf]

© 2021 Wiley-VCH GmbH

ADVANCED SCIENCE
Open Access

Supporting Information

for *Adv. Sci.,* DOI: 10.1002/advs.202101129

Wireless Soft Scalp Electronics and Virtual Reality System for Motor Imagery-based Brain-Machine Interfaces

*Musa Mahmood, Shinjae Kwon, Hojoong Kim, Yun-Soung Kim, Panote Siriaraya, Jeongmoon Choi, Boris Otkhmezuri, Kyowon Kang, Ki Jun Yu, Young C. Jang, Chee Siang Ang, and Woon-Hong Yeo**

Supporting Information for

Wireless Soft Scalp Electronics and Virtual Reality System for Motor Imagery-based Brain-Machine Interfaces

*Musa Mahmood, Shinjae Kwon, Hojoong Kim, Yun-Soung Kim, Panote Siriaraya, Jeongmoon Choi, Boris Otkhmezuri, Kyowon Kang, Ki Jun Yu, Young C. Jang, Chee Siang Ang, and Woon-Hong Yeo**

M. Mahmood[†], S. Kwon[†], H. Kim, Y.-S. Kim, Prof. W.-H. Yeo
George W. Woodruff School of Mechanical Engineering, College of Engineering, Georgia Institute of Technology, Georgia Institute of Technology, Atlanta, GA 30332, USA
Center for Human-Centric Interfaces and Engineering, Institute for Electronics and Nanotechnology, Georgia Institute of Technology, Atlanta, GA 30332, USA

J. Choi, Prof. Y.C. Jang
School of Biological Sciences, Georgia Institute of Technology, Atlanta, GA 30332, USA

P. Siriaraya, B. Otkhmezuri, Prof. C.S. Ang.
School of Computing, University of Kent, Canterbury, Kent CT2 7NT, UK

K. Kang, K.J. Yu
School of Electrical and Electronic Engineering, Yonsei University, Seoul 03722, Republic of Korea

Prof. W.-H. Yeo
Wallace H. Coulter Department of Biomedical Engineering, Parker H. Petit Institute for Bioengineering and Biosciences, Institute for Materials, Neural Engineering Center, Institute for Robotics and Intelligent Machines, Georgia Institute of Technology, Atlanta, GA 30332, USA
E-mail: whyeo@gatech.edu (W.-H. Yeo)

†These authors contributed equally to this work.

Section S1. Fabrication process of flexible microneedle electrodes.

1. Clean a negative PDMS microneedle array mold (MicroPoint, Singapore) with IPA and dry at 60 °C for 10 minutes.
2. Make a positive mold from the negative PDMS mold with epoxy resin (ExpoxAcast, Smooth On, Inc).
3. Make multiple PDMS (Sylgard 184, Dow Corning) negative molds from the positive epoxy resin mold.
4. Put the PDMS negative mold on a glass slide with a PDMS bonding layer between them.
5. Treat the PDMS negative mold with ambient air plasma for 2 minutes.
6. Apply a thin layer of Polyimide (PI) (PI 2610, HD Microsystems) on the negative PDMS mold by scraping with a razor blade.
7. Spin coat PI on the mold at 800 RPM for 60 seconds.
8. Soft-bake the PI at 100 °C for 10 minutes.
9. Hard-bake the PI at 200 °C for 1 hour.
10. Peel of the PI microneedle array (PI MNA) from the PDMS mold.
11. Sputter Cr/Au (5nm/200nm) on the top side of PI MNA.
12. Sputter Cr/Au (5nm/200nm) on the bottom side of PI MNA.

Section S2. Fabrication process of stretchable interconnectors.

The fabrication of a stretchable interconnector uses a micro-machining process with a femtosecond laser cutter (WS-Flex USP, OPTEC), which offers higher throughput and lower cost than the conventional microfabrication in a cleanroom. A substrate for the interconnector is prepared by an electron-beam evaporating Cr/Au (5nm/200nm) on a 2-mil PI film (200HPP-ST, DuPont). The metal-coated PI film is then laminated on a PDMS-coated PET film to hold the material during the laser cutting process. Once an array of stretchable interconnectors is patterned on the metal-coated PI film, excess materials other than the patterned interconnectors are manually peeled off from the PDMS-coated PET film. With water-soluble tape, the interconnectors are transfer-printed on a soft elastomer substrate (Ecoflex 00-30, Smooth-On, Inc.), and areas other than their contact pads are encapsulated with an additional layer of elastomer. The interconnectors are electrically connected to the electrode and sensor with silver paint (Fast Drying Silver Paint, Ted Pella).

Section S3. Optimized channel selection.

44-channels data out of 128 channels from 13 subjects were considered, as performed in the prior work (High-Gamma Dataset) (*1*). A flow-chart demonstrating the data flow process is shown in Supplementary Fig. S7. The full dataset is simply preprocessed using a 3rd-order Butterworth bandpass filter, with corner frequencies at 4 Hz and 30 Hz, and split into windows of 4 seconds (Fig. S7A). A convolutional neural network (CNN) with standard convolutions on the first layer, with a filter size of (10, 1), followed by four spatial convolutional layers, is used to train the full dataset (Fig. S7B). A generator then cycles through the data channels, eliminating the remaining channels in order to calculate the output perturbation on the selected channels (Fig. S7C). This data is then fed into the trained network, and the output perturbations are compared (Fig. S7D) with the true expected outputs to generate the relative perturbations for that channel (Fig. S7E). These relative perturbations are summed by the channel to generate a final perturbation value (Fig. S7F), for each of the channels to be compared and ranked, as demonstrated in (Fig. S7G). These electrode positions correspond with custom positions on the 128-channel system (waveGuard, ANT Neuro) used in the referenced paper (*1*). The electrode positions for our main study were based on analysis of this and other public motor imagery datasets, as well as some empirical experimentation to determine the optimal 6-electrode setup (Figure 1 in the main text). The setup was determined to reduce the number of electrodes and complexity of the setup as much as possible without significant reduction in classification performance. Table S1 summarizes PSDA and cubic SVM classification results.

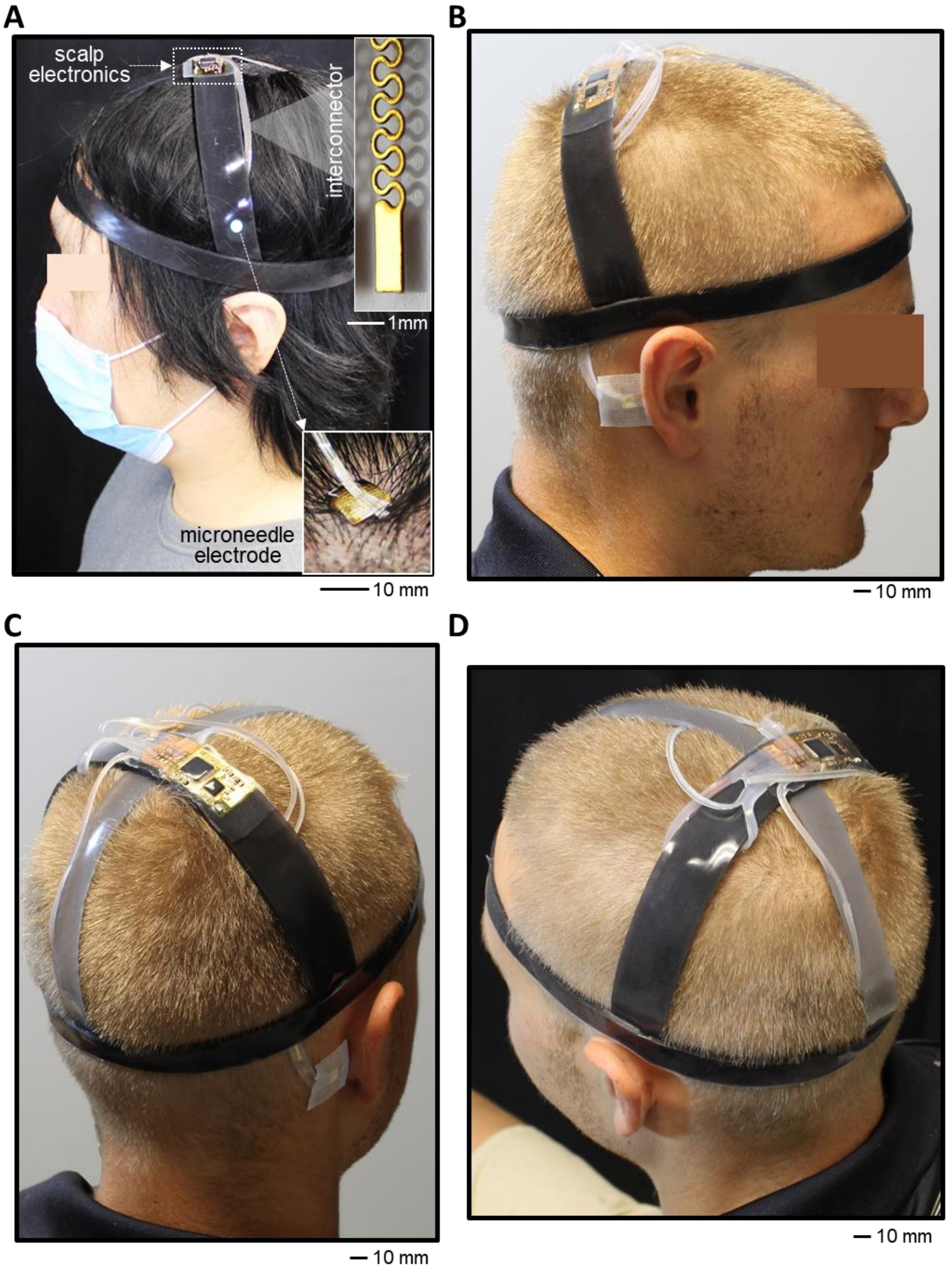

Figure S1. Overview of a wireless soft scalp system. (A) The wearable soft headset is composed of low-modulus elastomeric bands molded together for securing multiple FMNEs in place. The primary band wraps around the head about the axial plane with an attached membrane crossing from the forehead to the inion to secure the electrodes along that axis, while another membrane from ear to ear secures electrodes on the temporal lobes. Inset images show the stretchable interconnect (top right), and microneedle electrode attached to scalp (bottom right). (B-D) Additional angles of the complete headset setup.

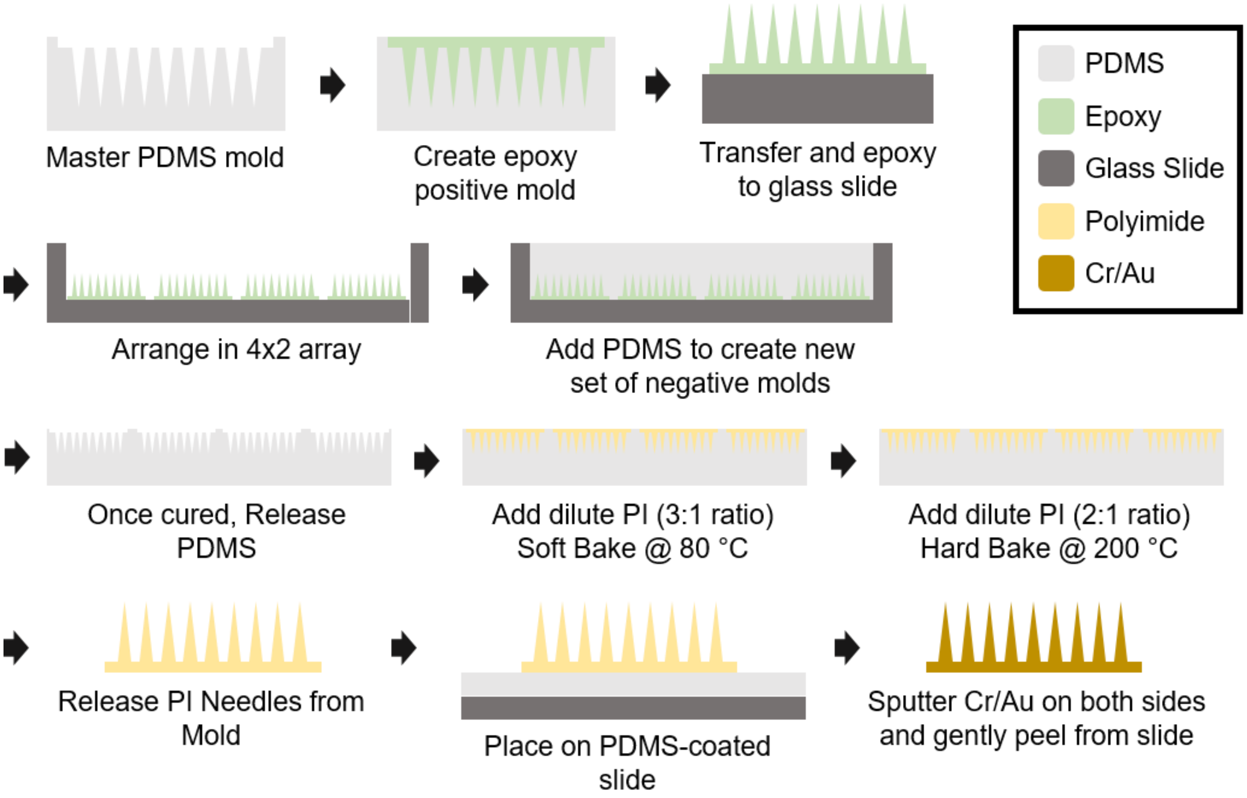

Figure S2. A fabrication process of an array of flexible microneedle electrodes.

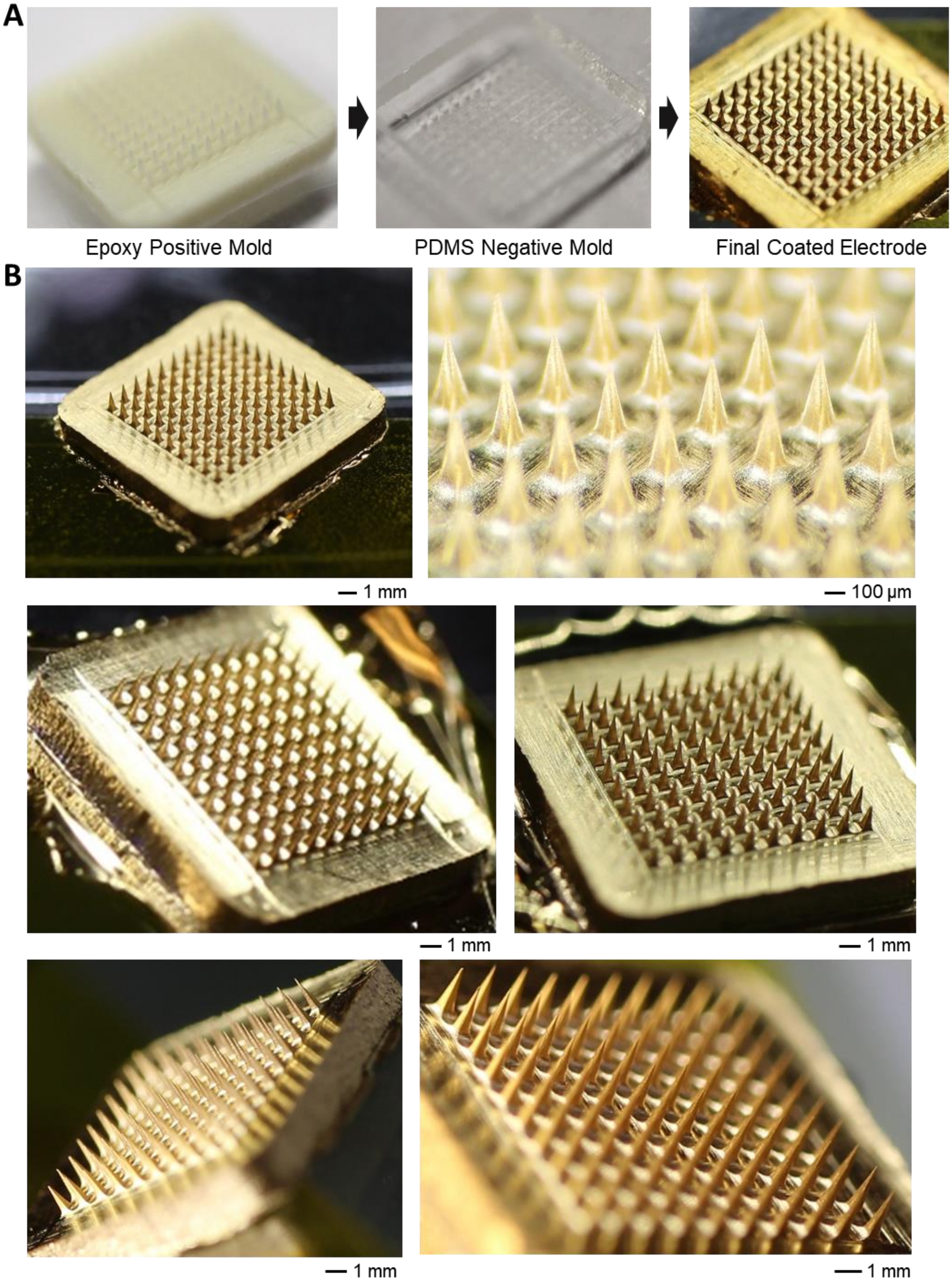

Figure S3. (A) Photos of electrode fabrication processes and (B) a collage of optical images of the finalized electrodes from various angles. The impedance and impedance density of microneedles are summarized in Table S2.

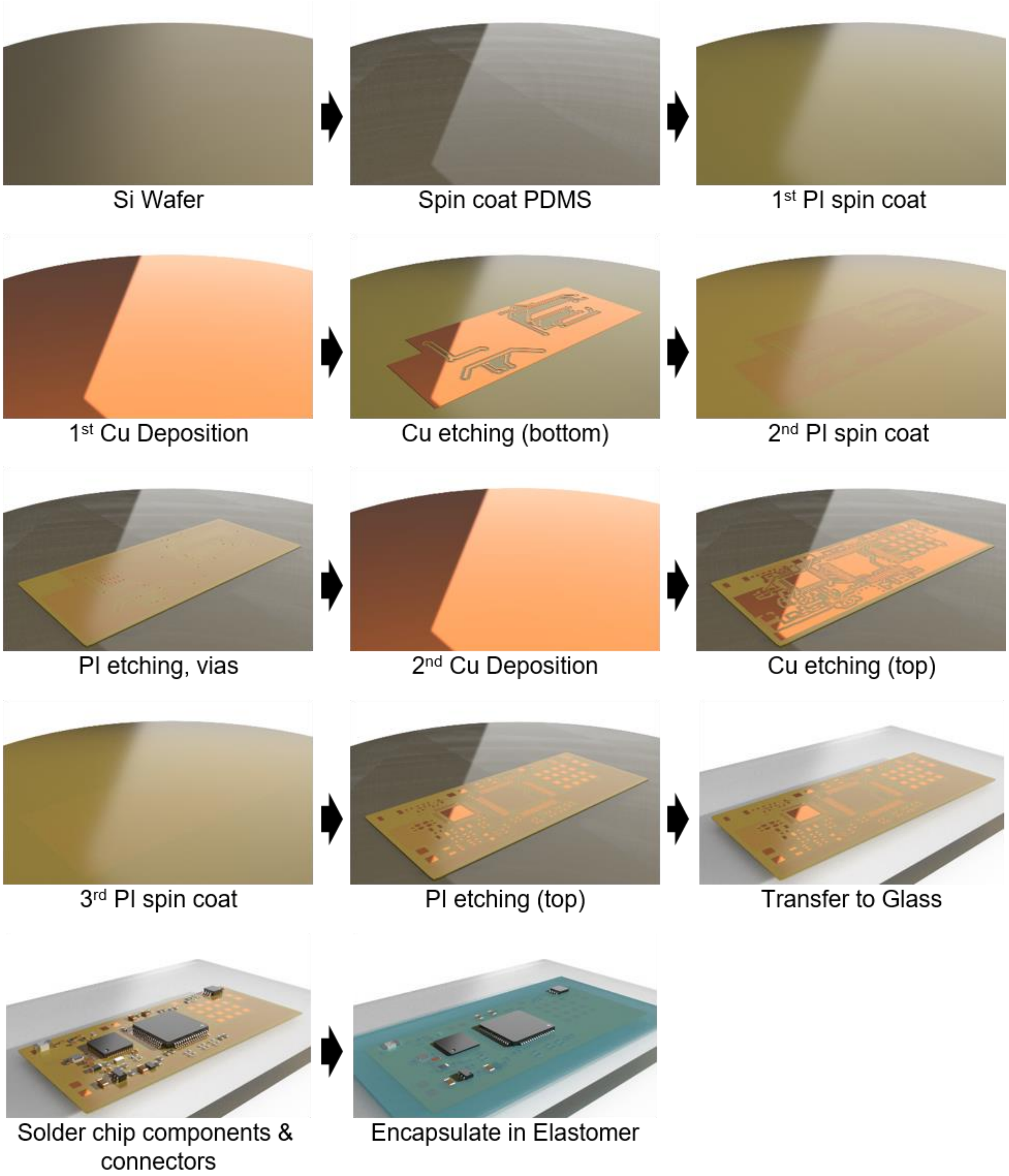

Figure S4. A fabrication process of a flexible wireless circuit.

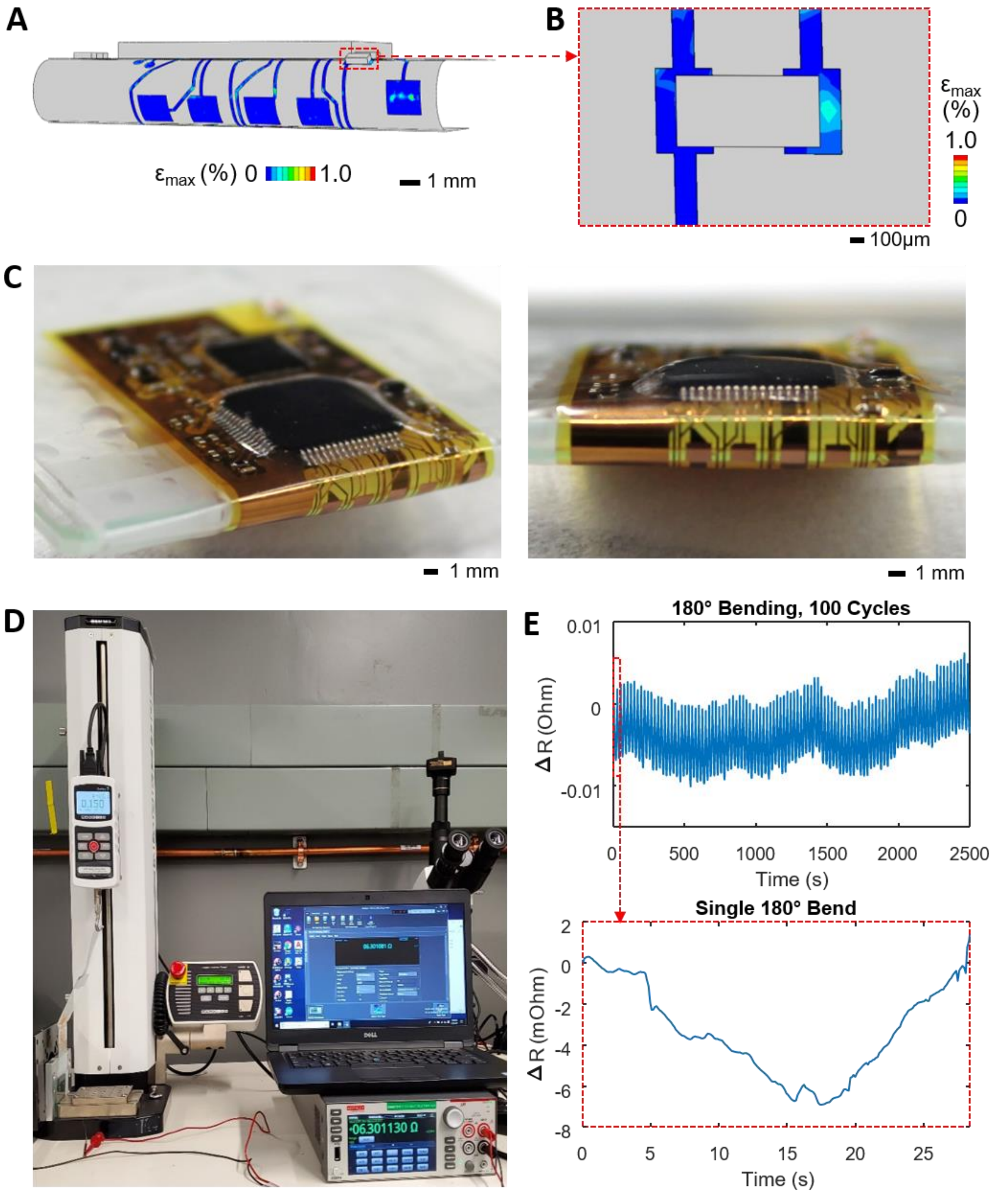

Figure S5. Mechanical characterization of a flexible circuit. (A) Computational mechanics modeling data of a circuit bent at the location with components and interconnects, demonstrating 180° bending with a radius of curvature of 1.5 mm. (B) Close-up area from (A). (C) Photos of a circuit undergoing bending by following the computational modeling. (D) Mechanical test stand setup for cyclic bending, with multimeter and laptop for measuring resistance. (E) Resistance data over 100 bending cycles (top), and a close-up of data over a single cycle (bottom), showing the minimal fluctuation of resistance.

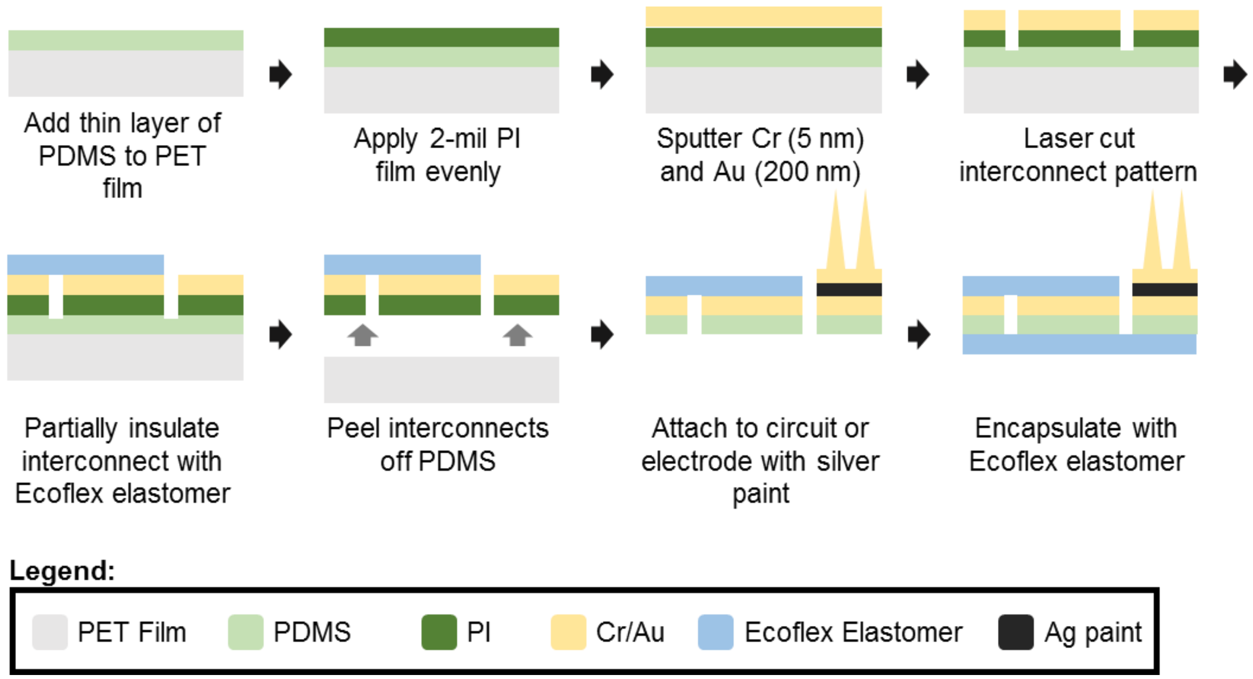

Figure S6. A fabrication process of a stretchable interconnector.

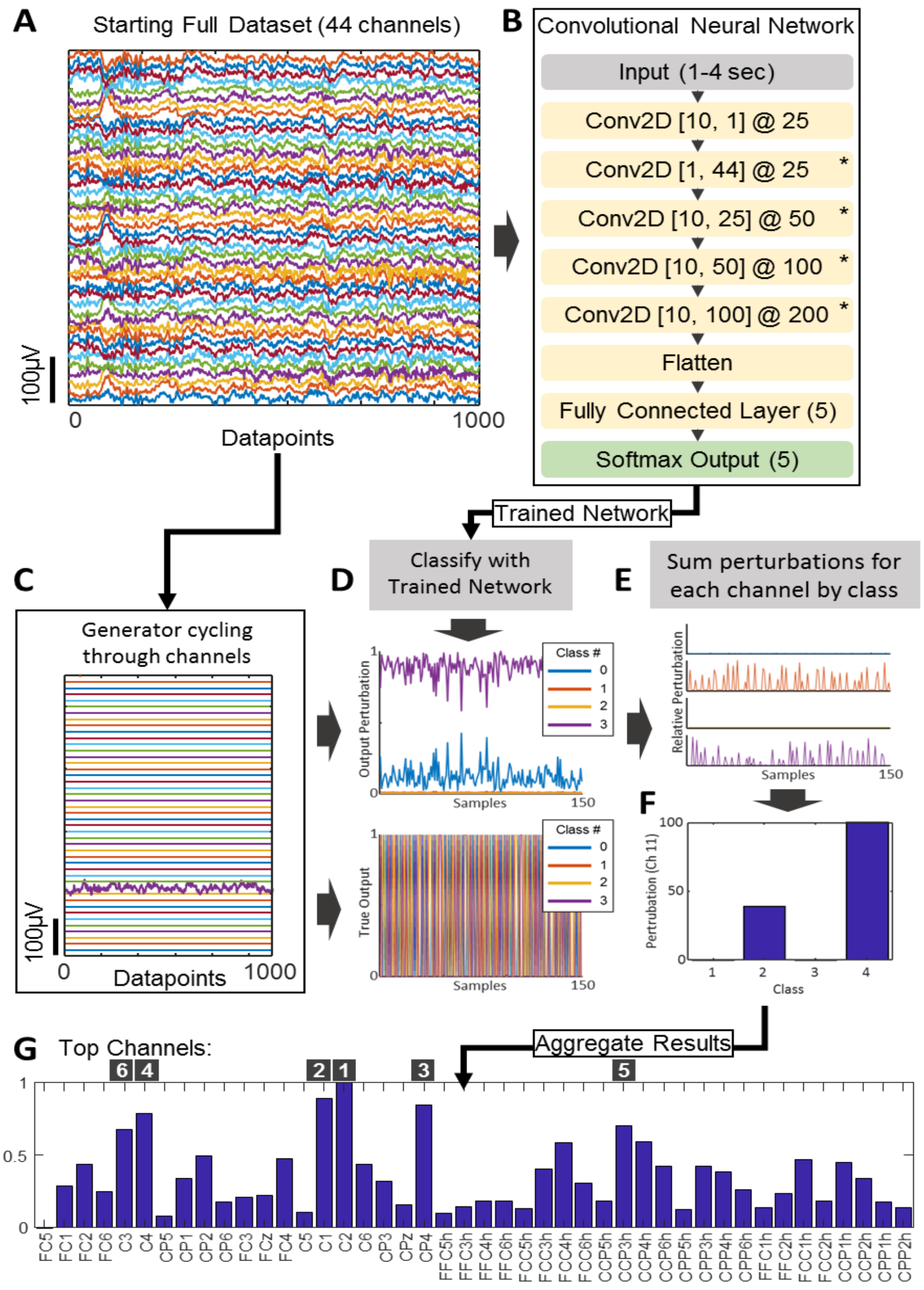

Figure S7. Optimized channel selection process. (A) A complete 44-channel MI dataset is (B) trained on a spatial-CNN model. (C) A generator is used to cycle through the channel, setting the remaining channels as inactive, and (D) passed through the trained classifier. (E) The output perturbations are then compared with the true outputs, resulting in the relative perturbation, (F) which is summed over the classes to get the per-class perturbations for each channel. (G) A bar chart of each channel's relative perturbations is shown with the top-6 channels labeled.

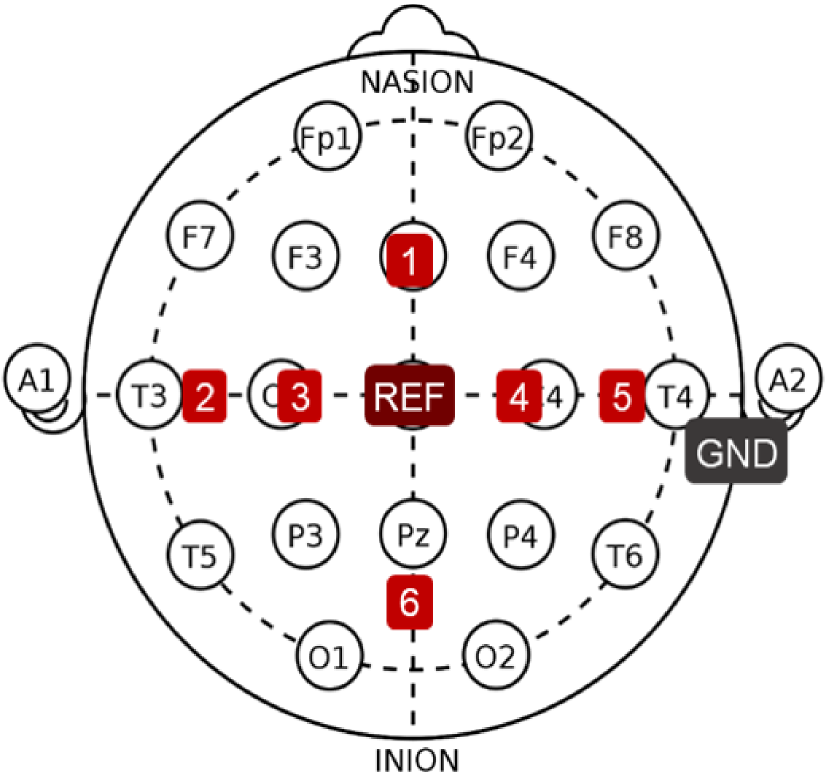

Figure S8. Locations of electrodes, including Fz, C5, C3, C4, C6, and POz, with the reference electrode at Cz, and the ground electrode placed at the mastoid.

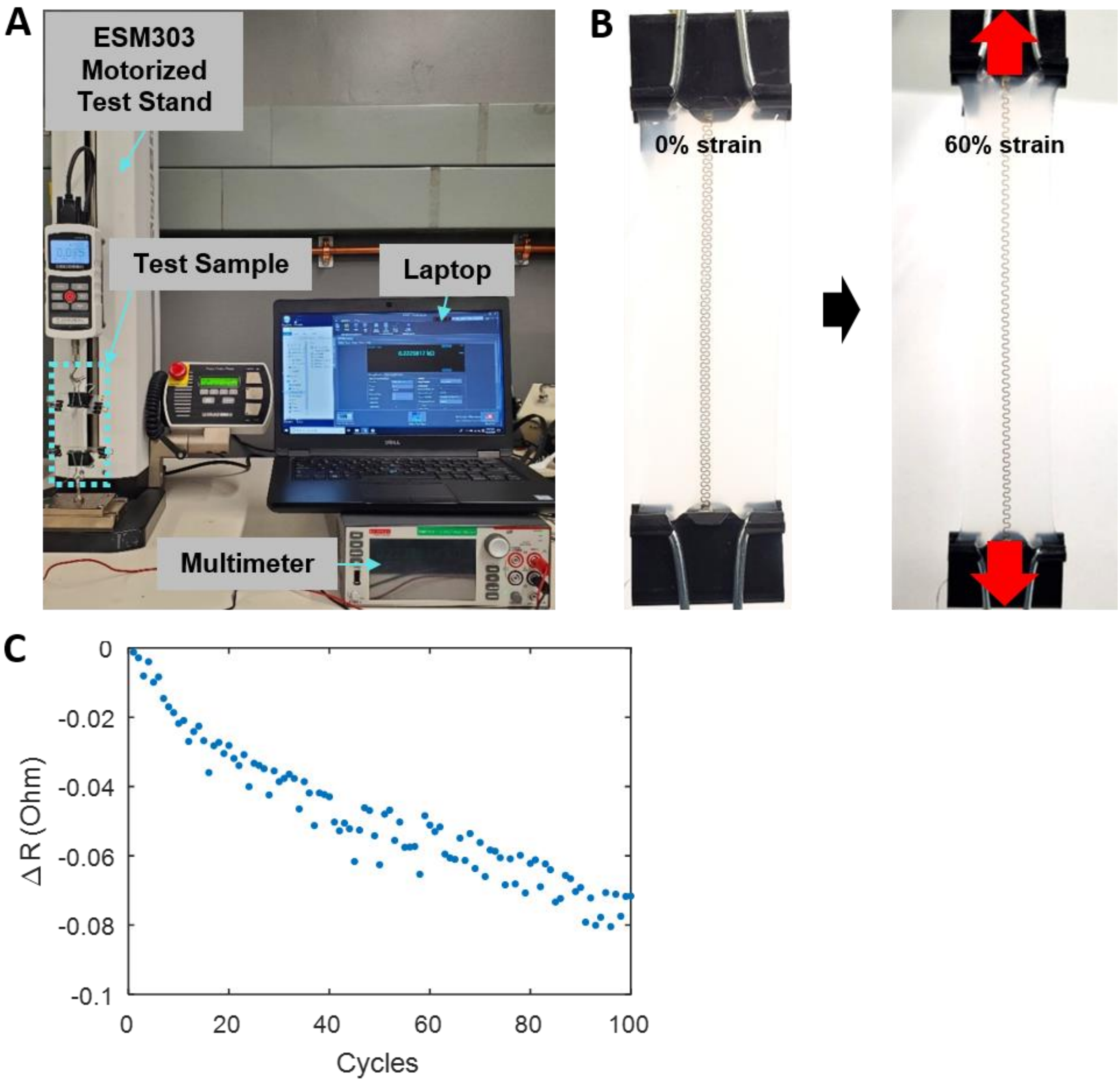

Figure S9. Experimental setup of a stretching test. (A) Motorized test stand (ESM303, Mark-10), multimeter, and laptop data acquisition setup for stretch testing of stretchable interconnects. (B) A two-inch segment of stretchable interconnects secured between two binder clips pre-strain (left), and at 60% strain (right). (C) Change in resistance over 100 stretching cycles with 60% strain.

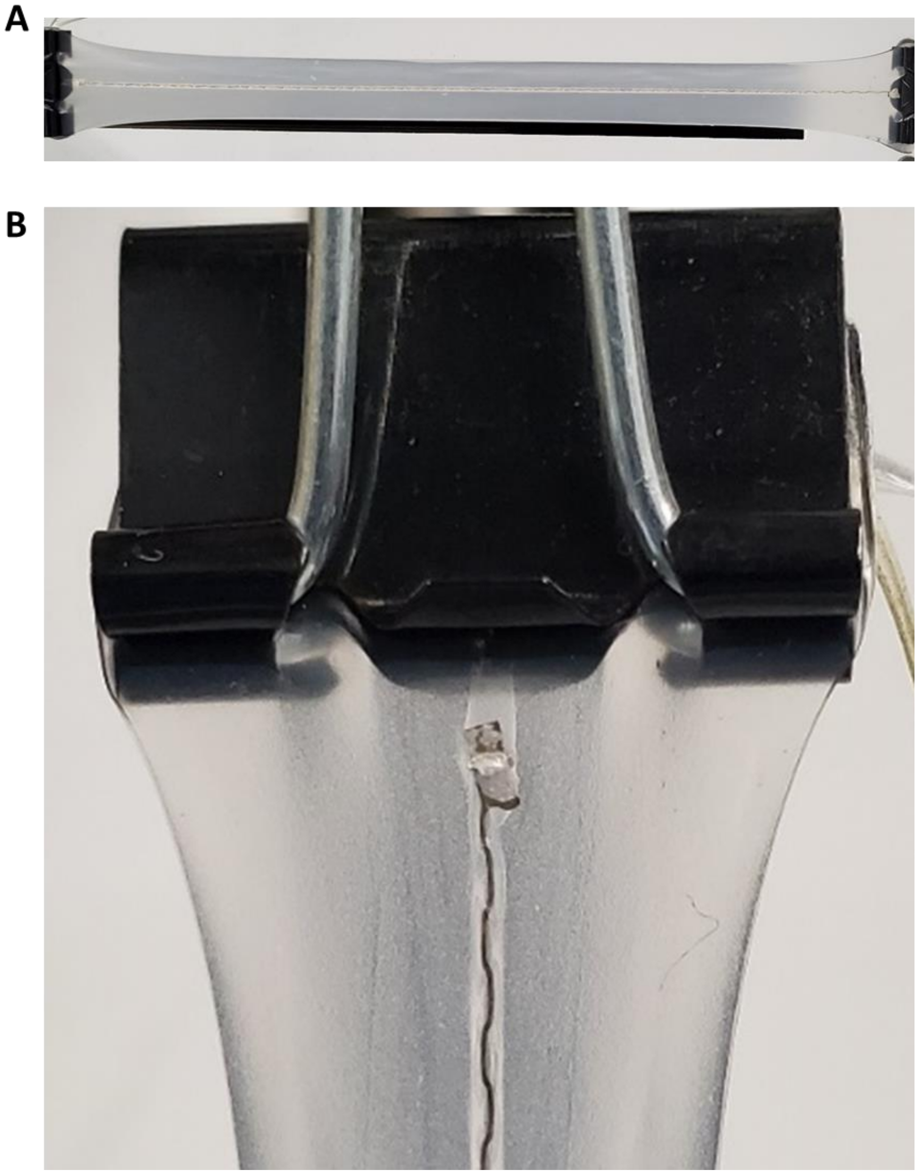

Figure S10. Close-up photos of a flexible interconnector with an excessive strain. (A) An entire view of an interconnector at 275% strain. (B) Close-up of the failure point near the contact pad.

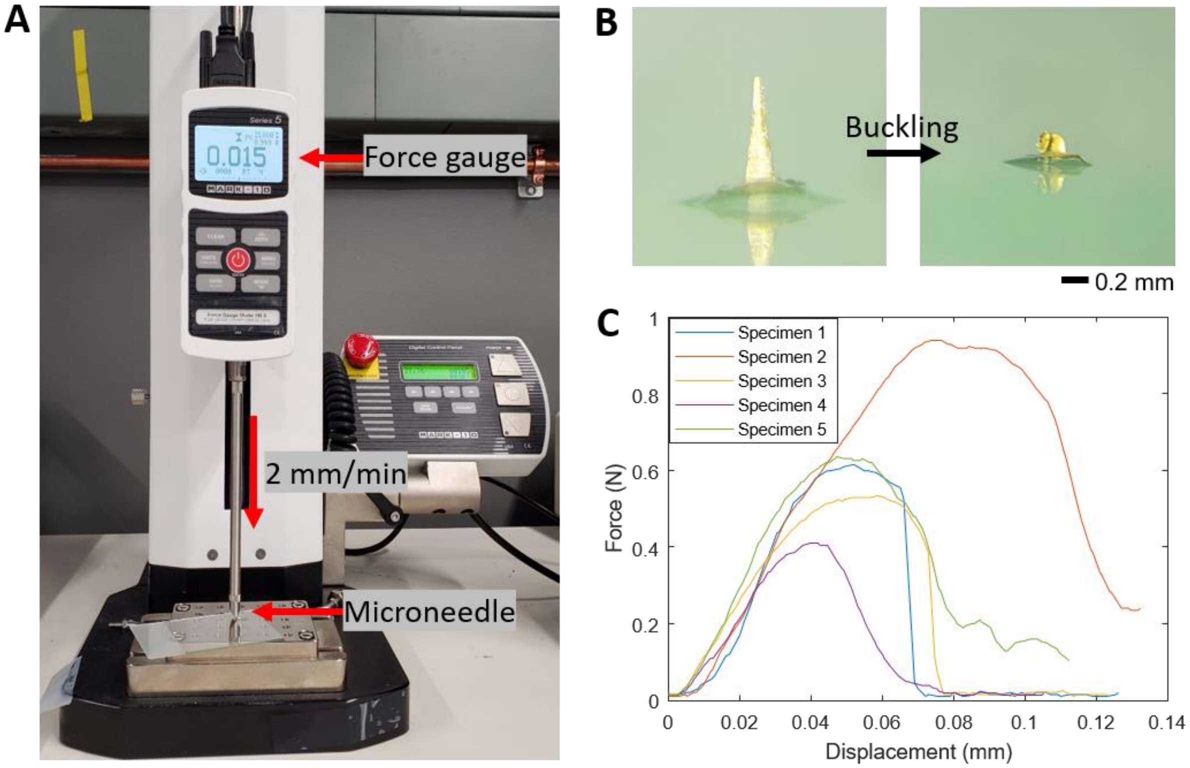

Figure S11. Buckling force evaluation of a single microneedle electrode. (A) Buckling force testing setup in which the motorized force gauge applies an axial force upon a single microneedle electrode and measures the axial force until its fracture. (B) Photos of a single microneedle before (left) and after (right) the buckling test. (C) The buckling test data of five different single microneedle test specimens where buckling fracture begins at the peak force value.

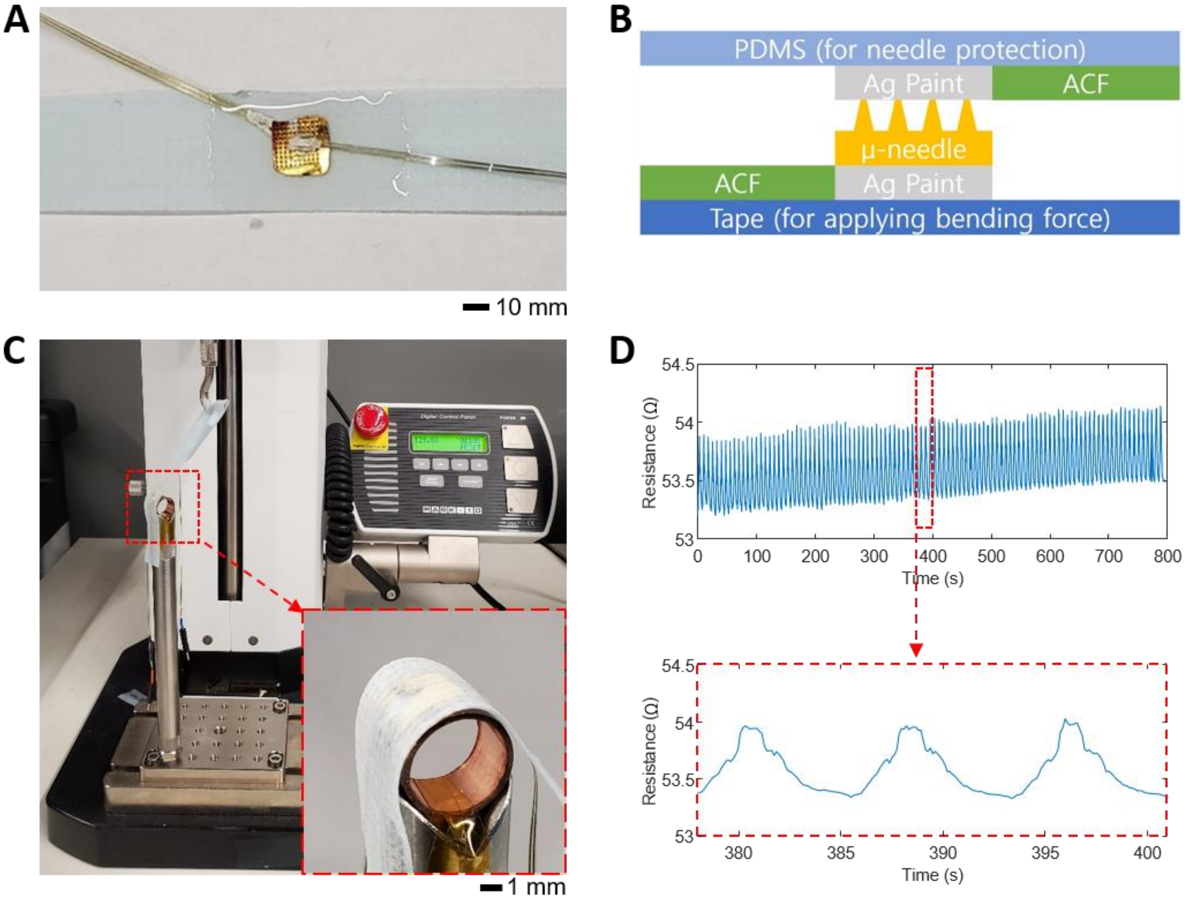

Figure S12. Flexibility evaluation of a microneedle electrode. (A) Photo of a test specimen structure for microneedle electrode cyclic bending test. (B) Schematic of a cross-section of the test specimen. (C) Picture of cyclic bending test setup in which the motorized testing rig continuously bends and un-bends the test specimen over a cylinder with a 5 mm bending radius. (D) Resistance data over 100 bending cycles (top), and a close-up of data over three cycles (bottom), showing the minimal fluctuation of resistance in the microneedle electrode.

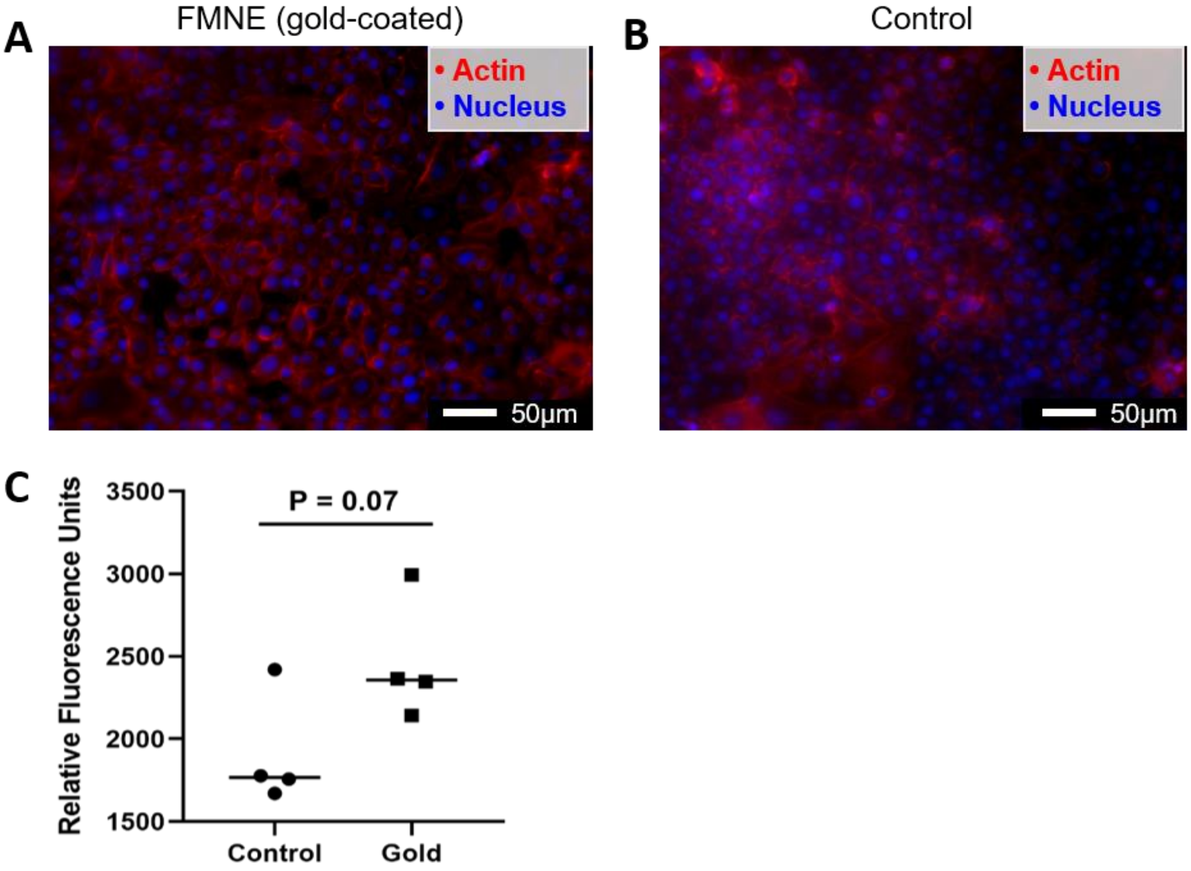

Figure S13. Cytotoxicity results. (A) Immunofluorescent Hoescht labeling of human keratinocyte cells after 5-day culture experiment in contact with Gold-coated FMNA electrode. (B) Control experiment data of immunofluorescent Hoescht labeling of human keratinocyte cells after 5-day culture. (C) Fluorescence measurements that quantify cytotoxicity with cell viability reagent for FMNA electrodes vs. control.

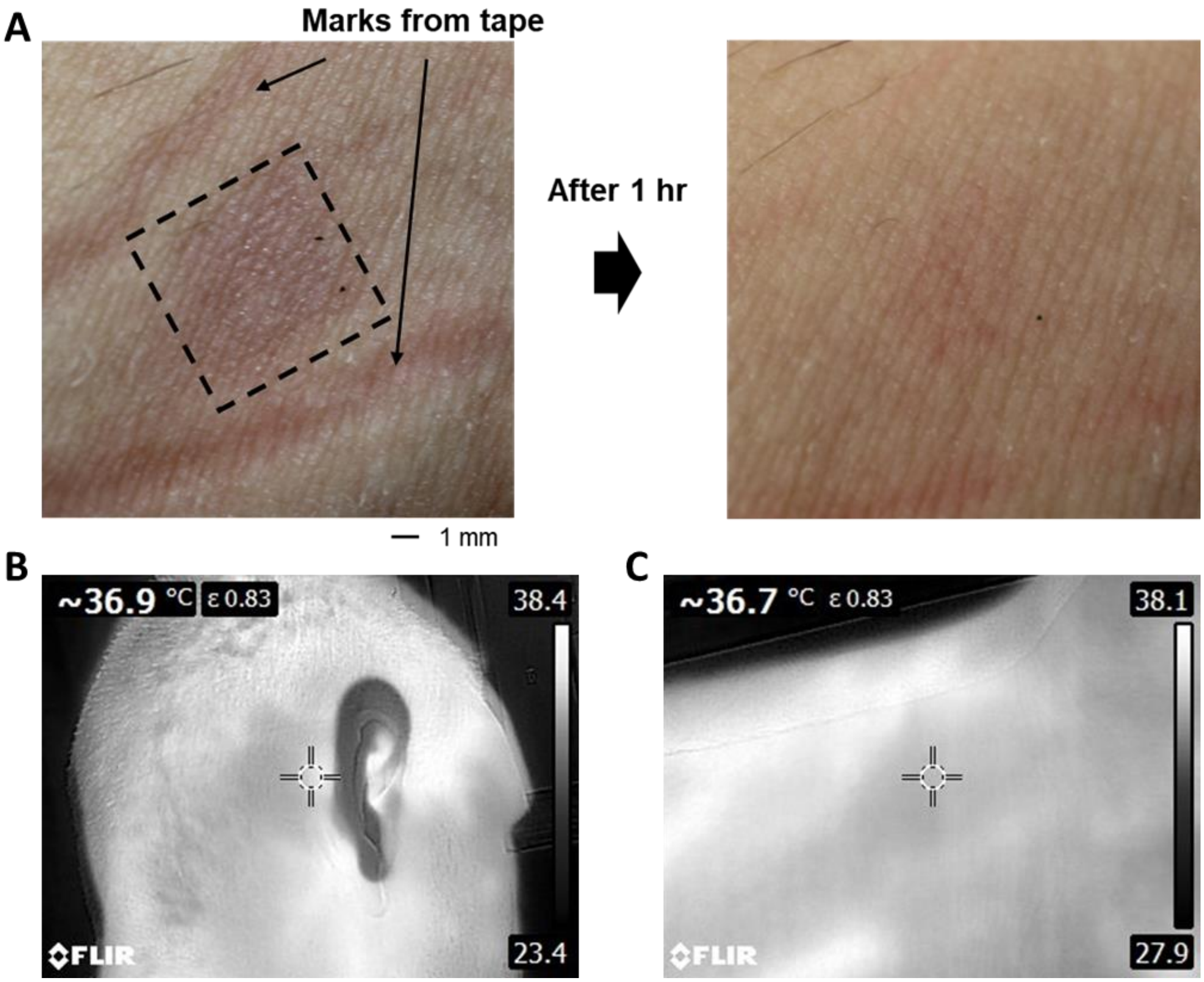

Figure S14. Microneedle electrode wearing results. (A) Photo of redness on skin after 48-hours continuous wear of gold flexible microneedle electrode (left), and fading redness after 1-hour post-release (right). (B) Thermal image (FLIR) showing skin temperature after 1 hour of wearing microneedle electrode behind the ear. (C) Thermal image showing skin temperature after 48 hours of wearing microneedle electrode on the skin (wrist).

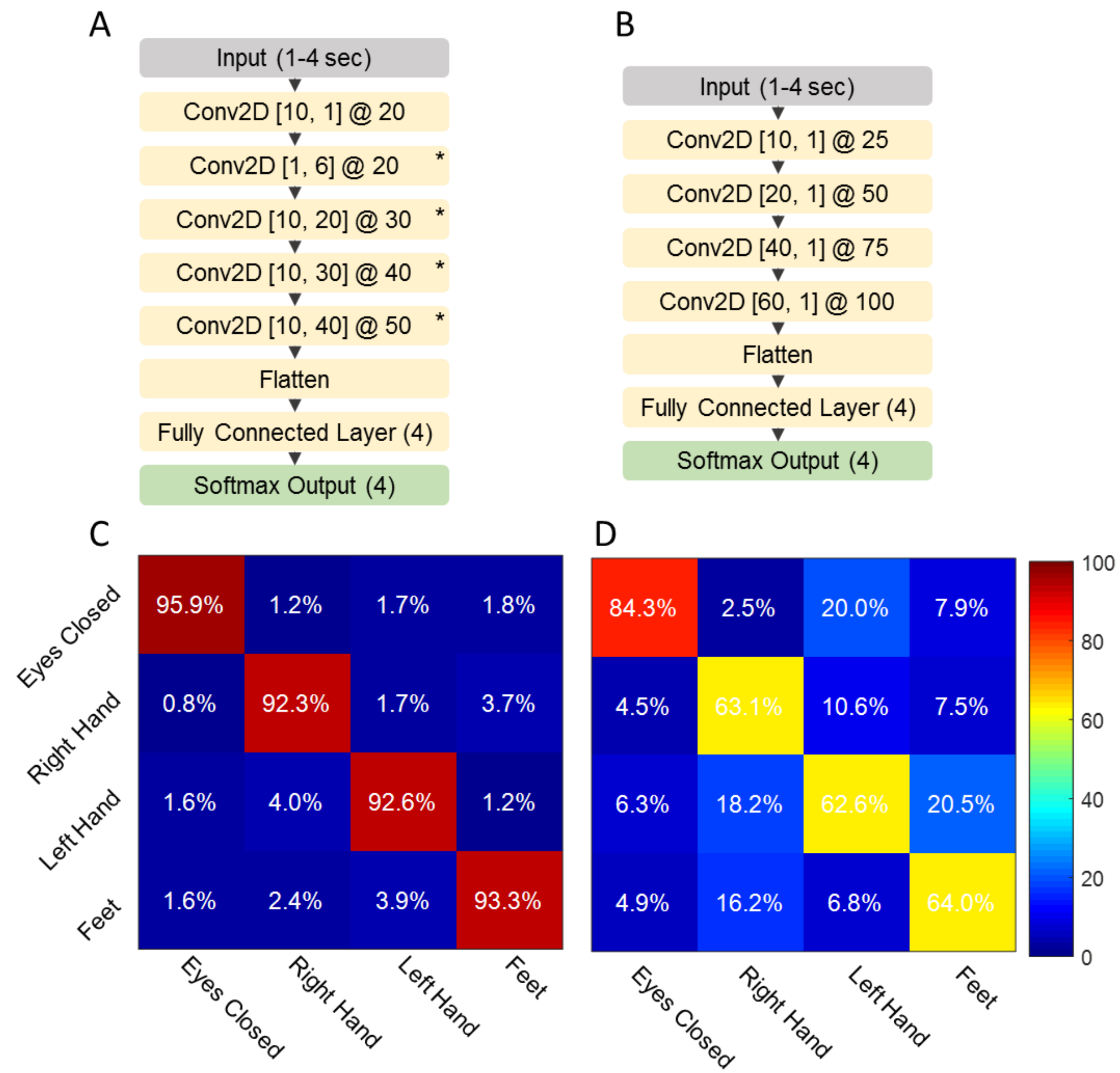

Figure S15. Comparison of CNN architectures and performance. (A) CNN architecture featuring multiple spatial convolution operations (Spatial-CNN), indicated with a '*' symbol. (B) Standard CNN architecture with normal convolution operations along with the time-domain signals (Standard-CNN). (C) Confusion matrix demonstrating Spatial-CNN classifier performance on the FMNE dataset (n = 2240 samples from 4 subjects, 560 samples per subject, window length w = 4 s). (D) Confusion matrix demonstrating Standard-CNN classifier performance on the FMNE dataset (n = 2240 samples from 4 subjects, 560 samples per subject, window length w = 4 s).

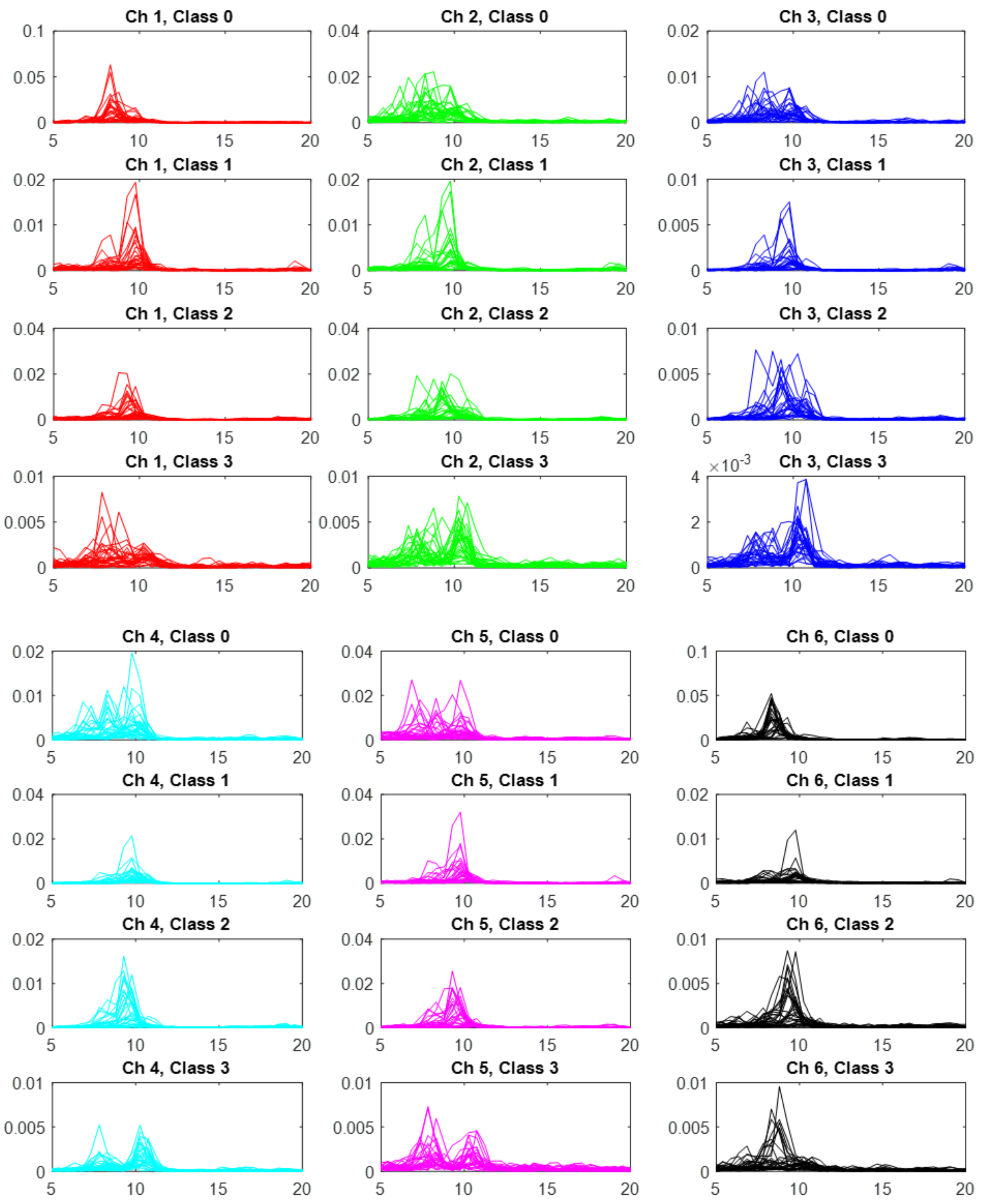

Figure S16. PSDA feature comparison of four EEG classes, demonstrating the presence of ERS/ERD in the form of power spectrum shifts for many of the samples shown (n=112 samples).

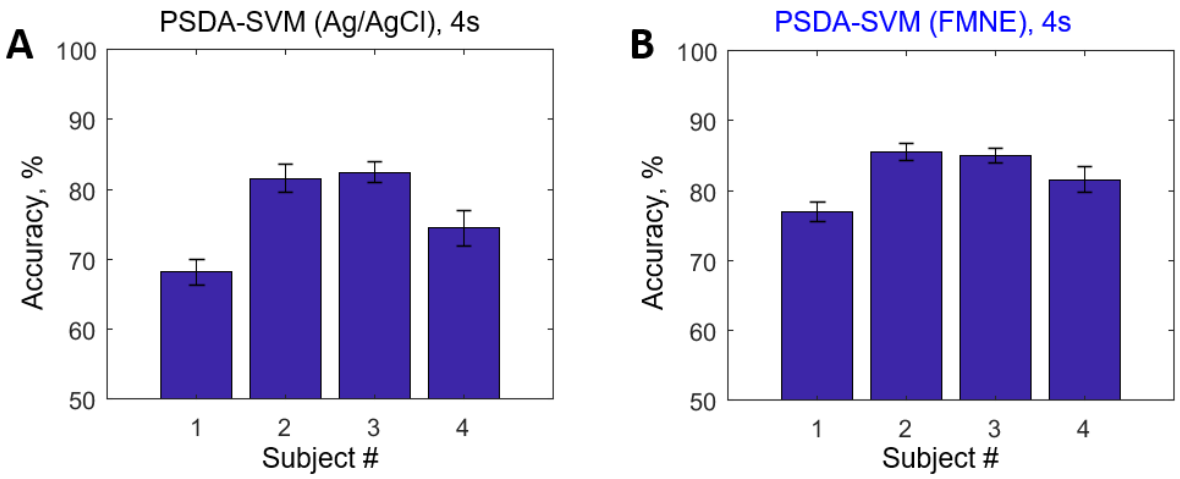

Figure S17. Comparison of classification results with PSDA and SVM with a cubic kernel function on conventional Ag/AgCl electrodes and FMNEs, showing a better accuracy from the FMNE dataset (4-second windows).

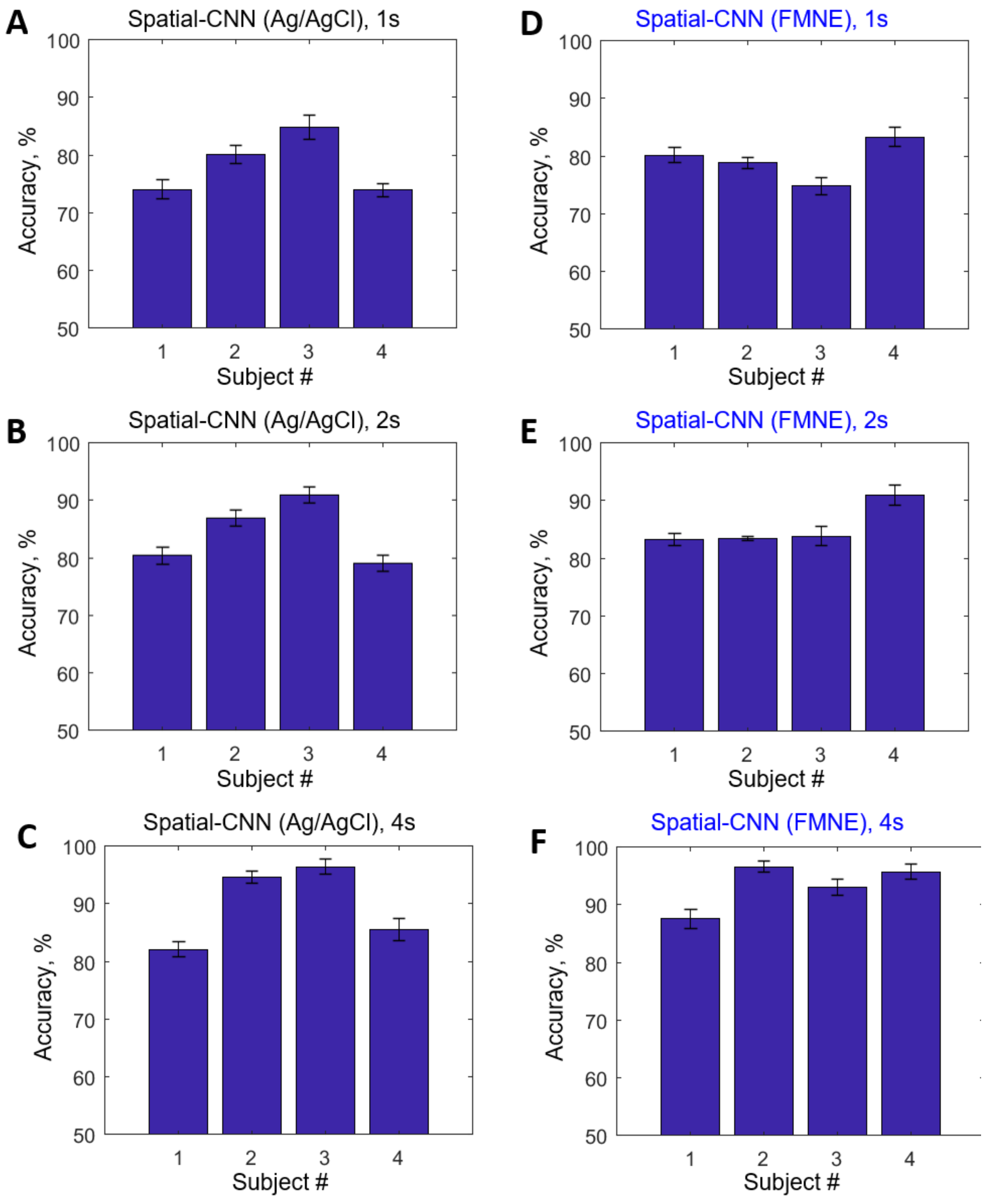

Figure S18. Comparison of the performance between Ag/AgCl electrodes and FMNEs across multiple window lengths (1, 2, and 4 seconds).

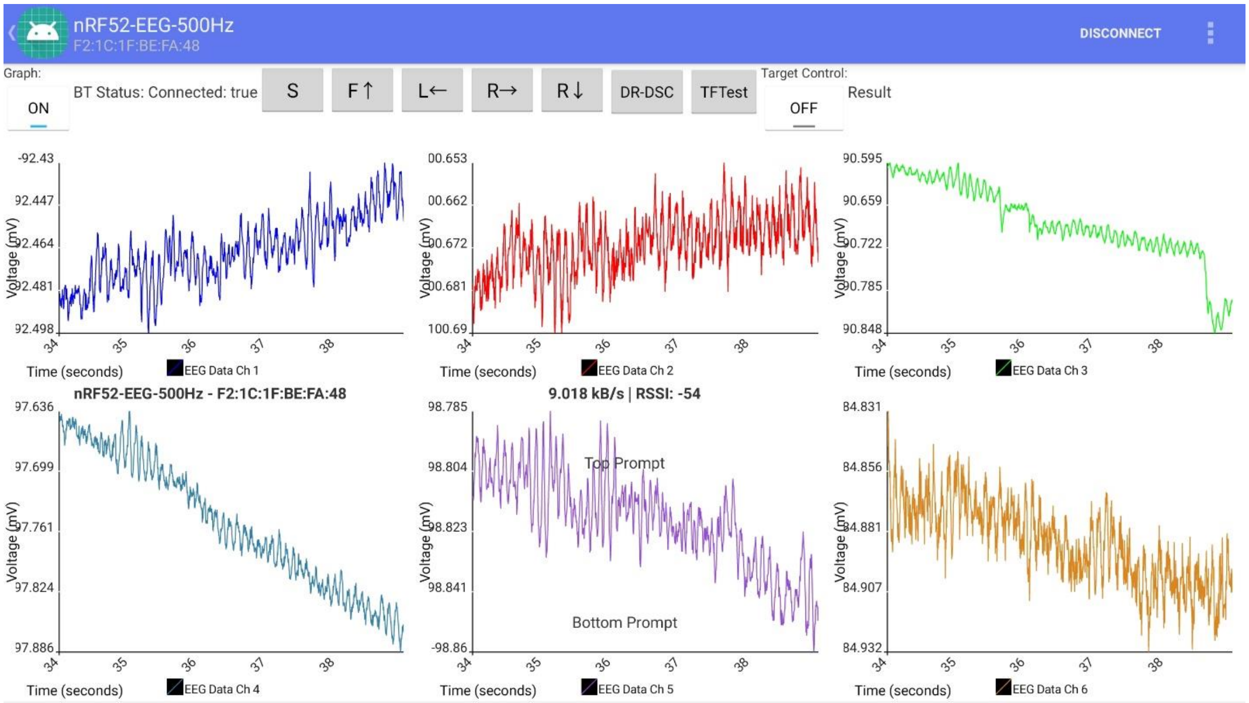

Figure S19. Android interface demonstrating real-time data plotting.

Table S1. PSDA and Cubic SVM Classification Results, Ag/AgCl with Conductive Paste Electrodes, 4-second time windows.

Subject	Fold 1	Fold 2	Fold 3	Fold 4	Fold 5	Average	Standard Error
1	66.67%	73.72%	61.54%	70.51%	68.59%	68.21%	1.82%
2	83.97%	81.41%	85.90%	83.33%	73.08%	81.54%	2.00%
3	81.41%	83.20%	89.74%	80.13%	82.69%	83.43%	1.49%
4	80.40%	79.60%	72.44%	75.00%	64.74%	74.44%	2.53%
**Mean**						**76.90%**	**1.96%**

Table S2. Comparison of impedance and impedance density of microneedle (MN) electrodes of varying height, with fixed base width of 200 µm, and pitch of 500 µm (14 x 14 array).

Electrode	500 µm MN	600 µm MN	700 µm MN	800 µm MN	MVAP (Ag/AgCl) electrode	Flat gold film electrode
Area (mm^2)	49	49	49	49	96	49
Impedance (kΩ)	43.6	35.7	29.7	23.3	20.8	129.3
Impedance Density (kΩ-cm^2)	21.4	17.5	14.6	11.4	20.0	63.4

Supplementary Videos

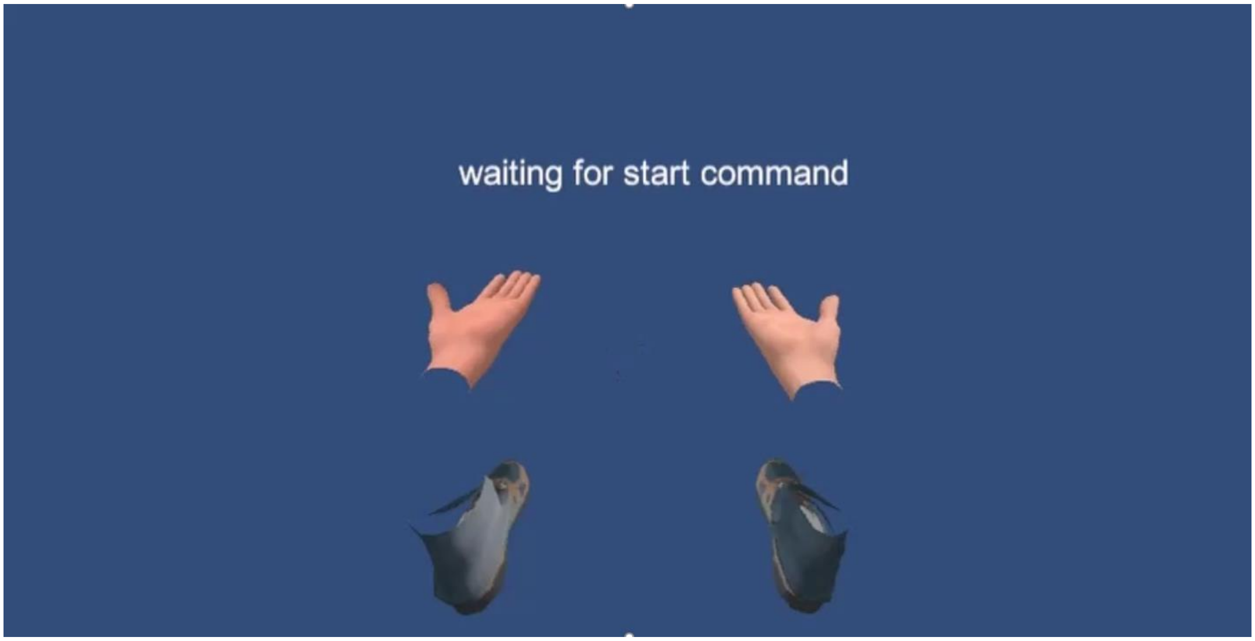

Video S1. VR training environment for MI-based machine interfaces.

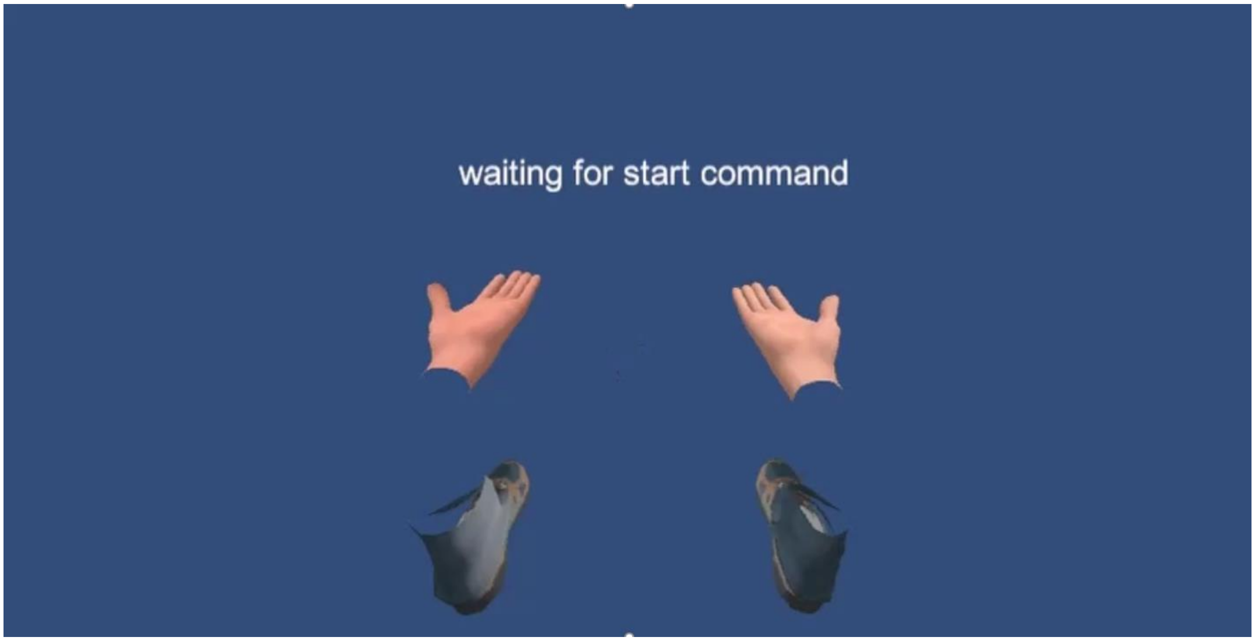

Video S2. VR real-time testing environment with randomized prompts.

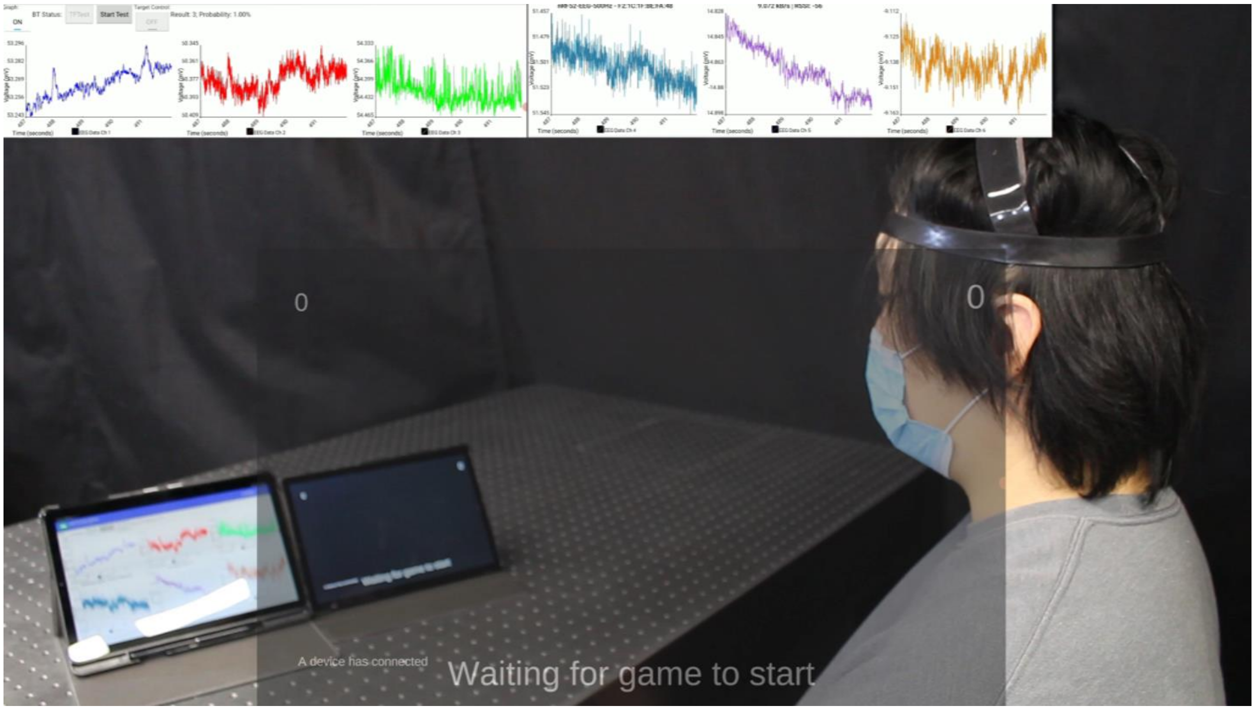

Video S3. Real-time demonstration of MI-based control of a VR game.

References:

1. R. T. Schirrmeister *et al.*, Deep learning with convolutional neural networks for EEG decoding and visualization. *Human brain mapping* **38**, 5391-5420 (2017).
